# Supplementary material for: Defects in t6A tRNA modification due to GON7 and YRDC mutations lead to Galloway-Mowat syndrome
Source: Nat Commun. 2019 Sep 3;10:3967. doi: 10.1038/s41467-019-11951-x (PMC6722078; doi:10.1038/s41467-019-11951-x)
Supplement: Supplementary file 1 — Supplementary Information [file 41467_2019_11951_MOESM1_ESM.pdf]

Supplementary Information

Defects in t^{6}A tRNA modification due to *GON7* and *YRDC* mutations lead to Galloway-Mowat syndrome

Arrondel *et al*.

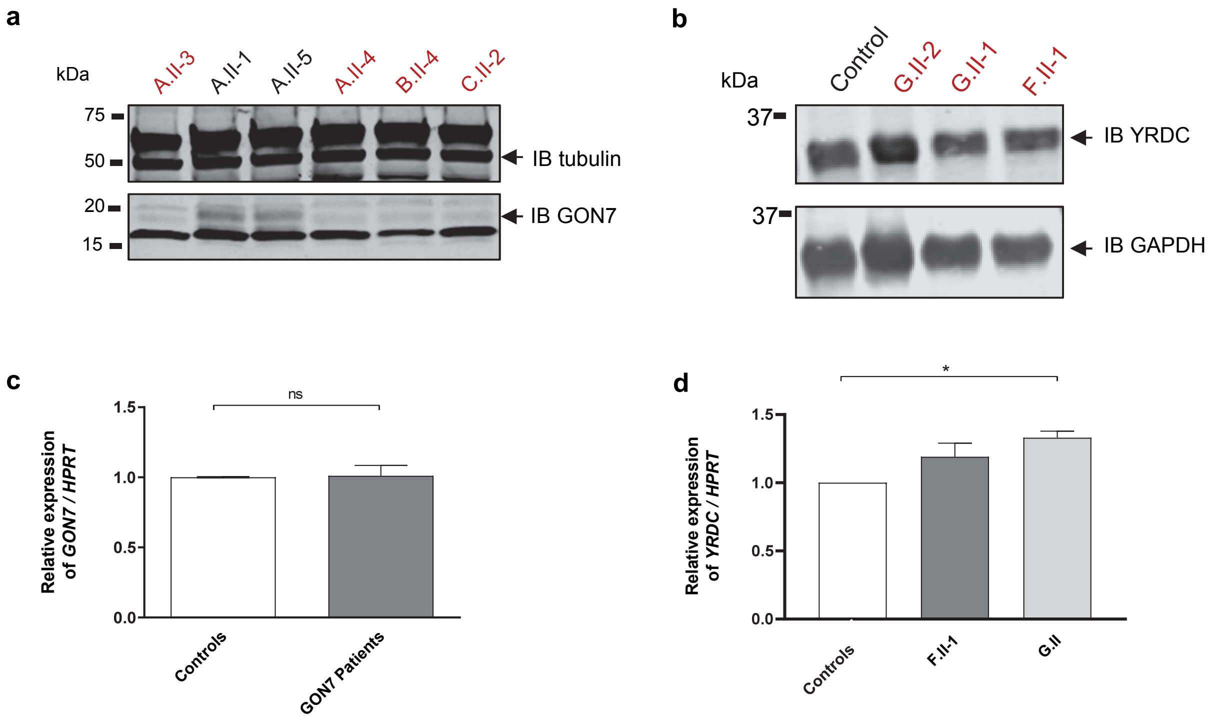

Supplementary Figure 1 | Expression of GON7 and YRDC in cells from controls and affected individuals. GON7 and YRDC protein expression levels were analyzed by western blot in lymphoblastoid cell lines (**a**) and in primary skin fibroblasts (**b**) from healthy children (in black) and affected individuals (in red) with the p.Tyr7* *GON7* mutation (a) or *YRDC* mutations (b). Tubulin and GAPDH were used as loading controls. (**c-d**) Relative expression of *GON7* and *YRDC* transcripts normalized to *HPRT* and compared to controls (mean ± s.e.m. of n=3 experiments, unpaired t-test in (c) and Kruskal-Wallis test followed by Dunn's *post-hoc* test in (d); ns=0.2751, *p =0.00137). Data correspond to the pool of 2 healthy individuals for the controls and 4 individuals with the p.Tyr7*GON7 mutation for *GON7* patients. For *YRDC* transcript expression, data of the 2 affected individuals from Family G have been pooled (G.II).

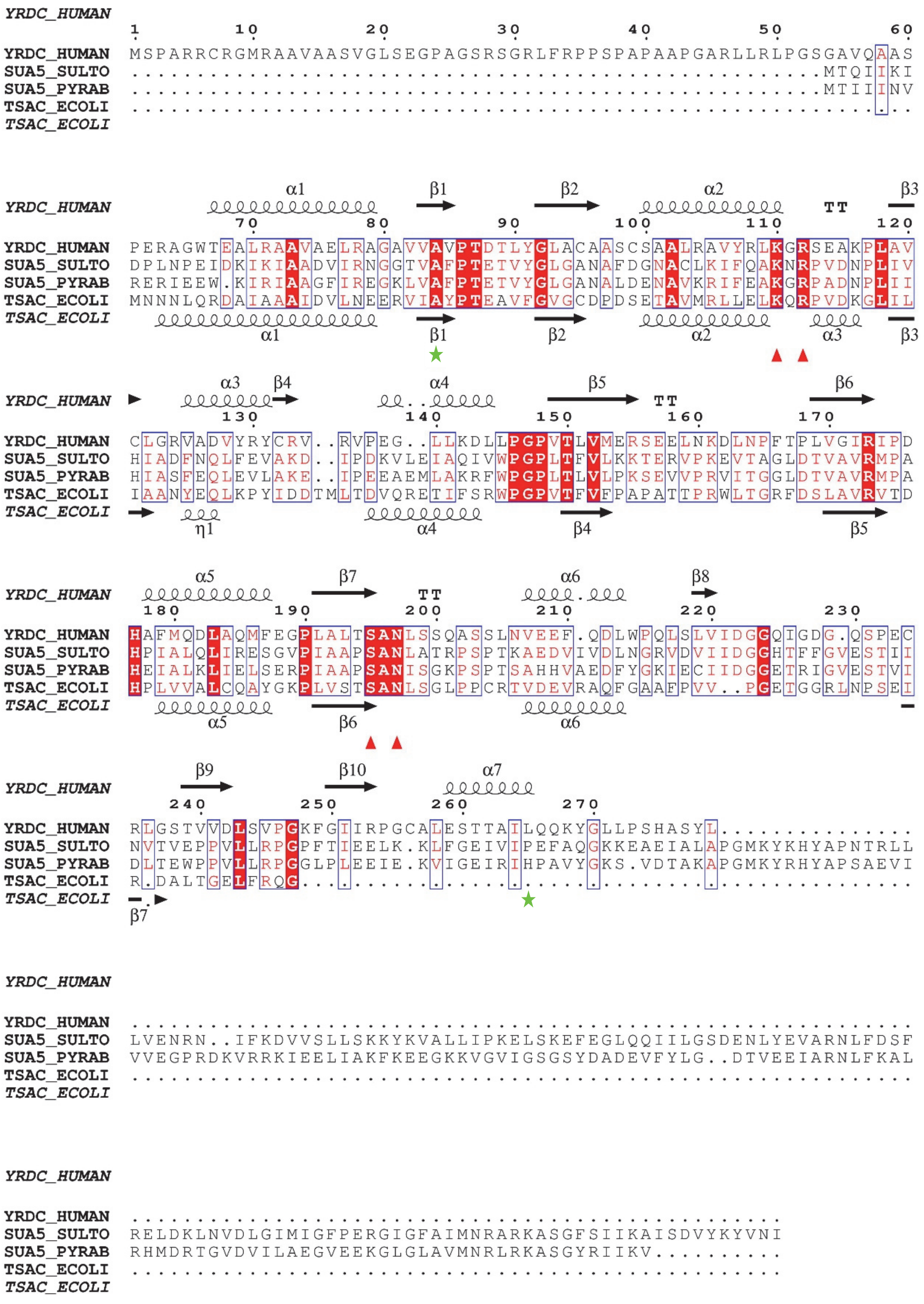

Supplementary Figure 2 | Structure based sequence alignment of YRDC/Sua5 sequences: human YRDC, Sua5 from *Sulfolobus tokadaii* and *Pyrococcus abysii*, *Escherichia coli* YrdC (TsaC). Crystal structure for the latter three are known. Sua5 proteins have an extra domain that is not present in the YrdC members of the TC-synthesizing enzyme family. Secondary structure elements extracted from the YRDC and *E.coli* YrdC structures are at the top and bottom, respectively. Red triangles represent amino acid of the active site. Positions Ala84 and Leu265 are marked by a green star.

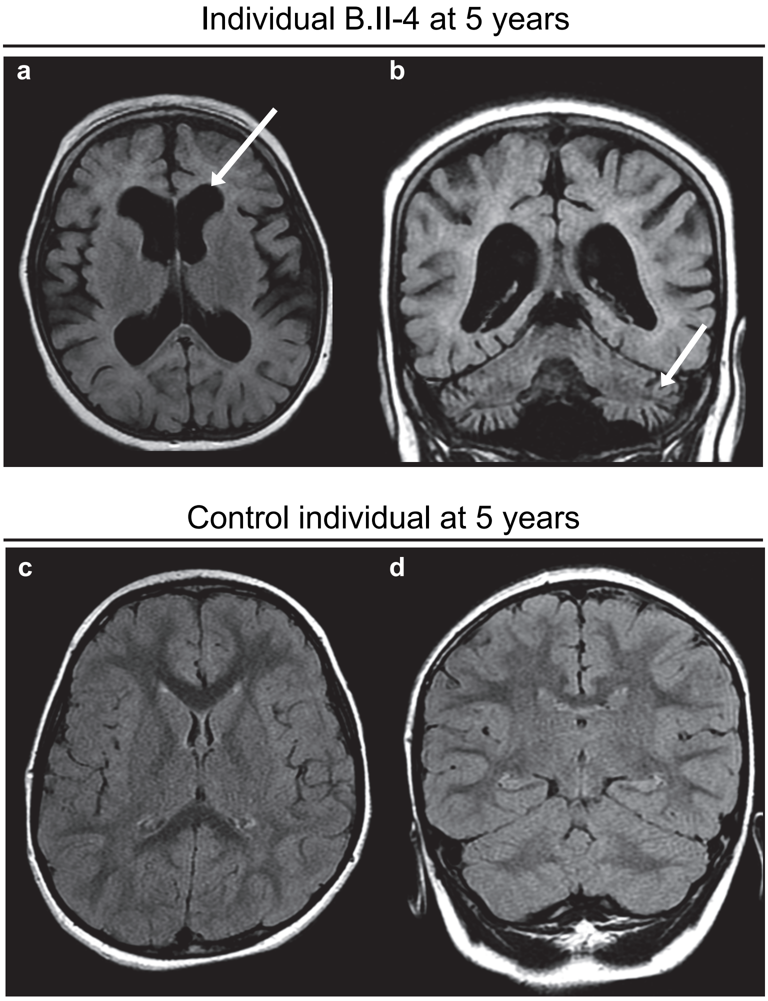

Supplementary Figure 3 | Brain anomalies in a *GON7*-mutated individual. Brain MRI of individual B.II-4 with *GON7* mutation at 5 years (**a-b**) compared to a control individual **(c-d).** The Flair (a) and coronal Flair (b) weighted images show ventricular dilatation and cerebellar atrophy (white arrows), respectively. (**c-d**) Brain MRI of a control individual at 5 years showing corresponding Flair (c) and coronal Flair (d) weighted images.

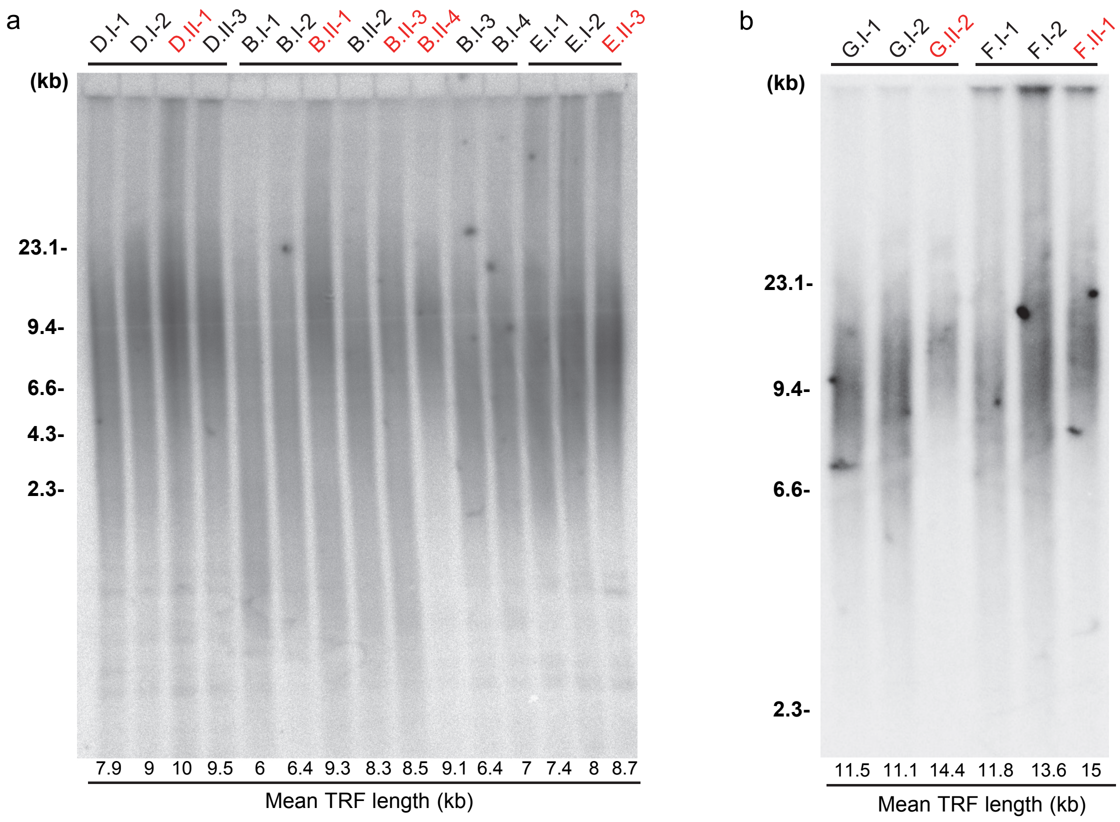

Supplementary Figure 4 | Analysis of telomere length in individuals with mutations in *GON7* and *YRDC* and their parents. Using the telomere restriction fragment (TRF) assay, the mean telomere lengths of whole blood cells from *GON7* (**a**) and *YRDC* (**b**) affected individuals and their healthy parents were estimated. This Southern blot shows a normal telomere length in affected children, with as expected, a telomere length higher than that of their parents. Genomic DNA was extracted from leukocytes.

a

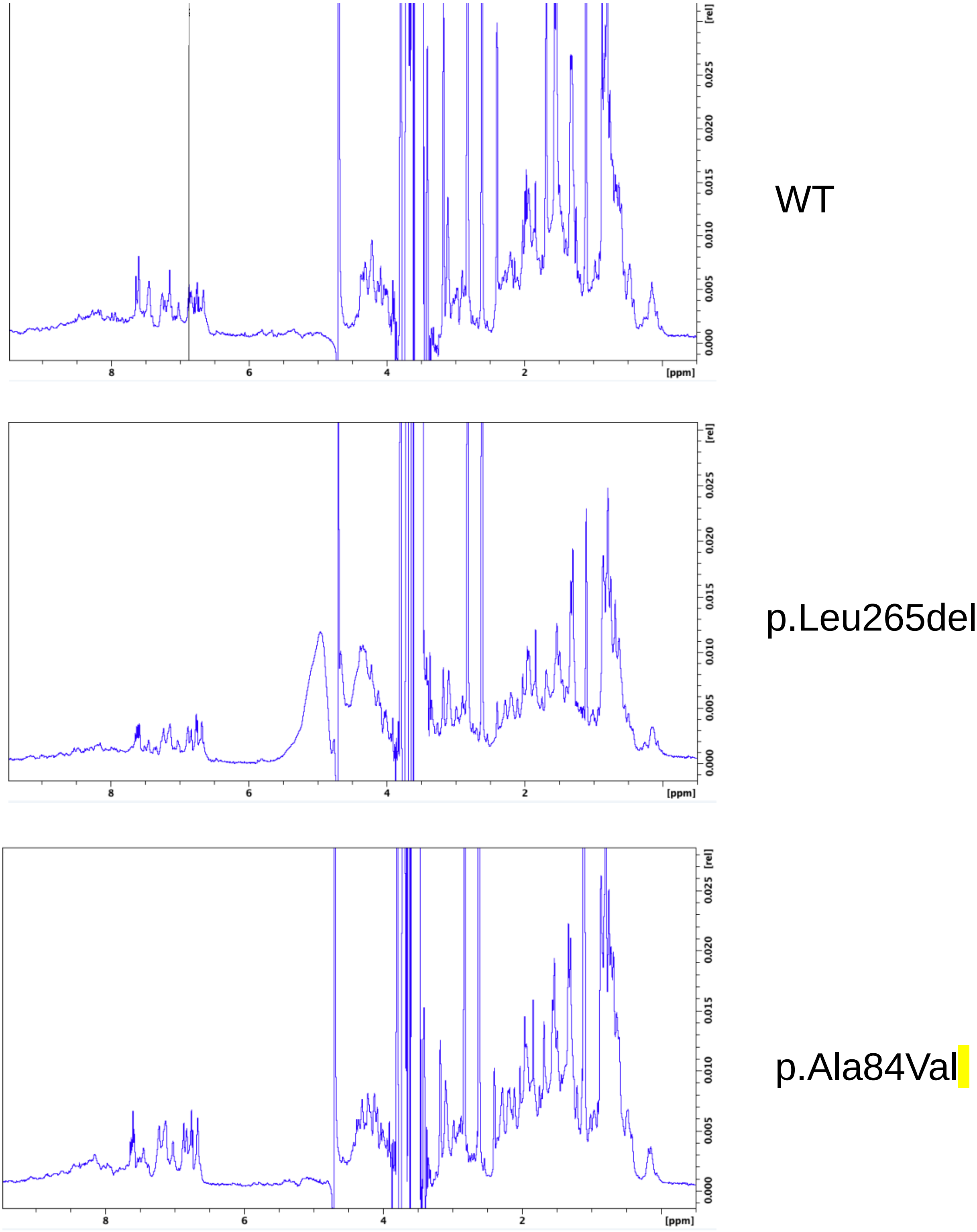

Supplementary Figure 5 | 1D 1H spectra and *in vitro* enzymatic activities of YRDC WT and mutants proteins. (**a**) 1D 1H spectra. (**b**) *In vitro* enzymatic activities. Numbers above histograms indicate mean values in mUA/min from triplicate measurements (+/- standard deviation). Pre-mix: assay buffer containing all substrates and PPiase enzyme but without YRDC.

b

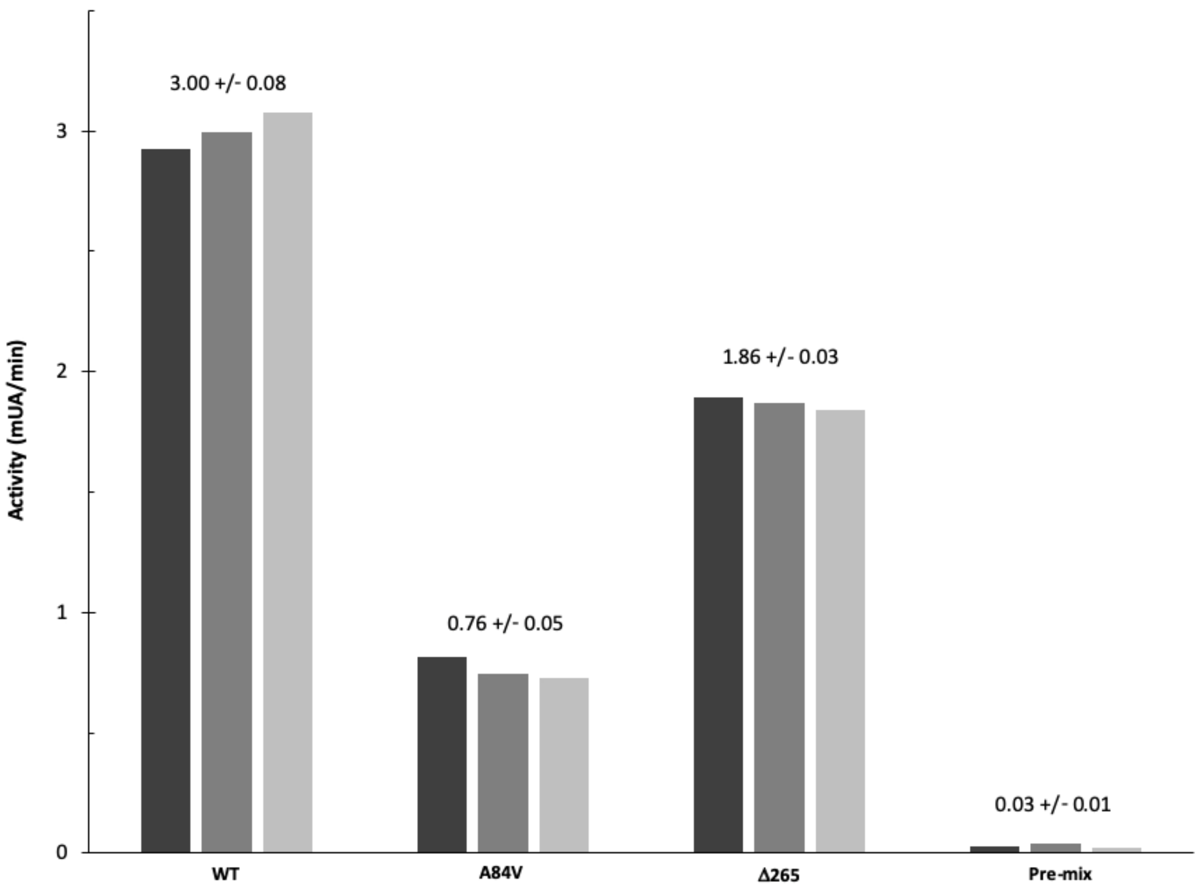
3.00 +/- 0.08
1.86 +/- 0.03
0.76 +/- 0.05
0.03 +/- 0.01
Activity (mUA/min)
0
1
2
3
WT
A84V
Δ265
Pre-mix

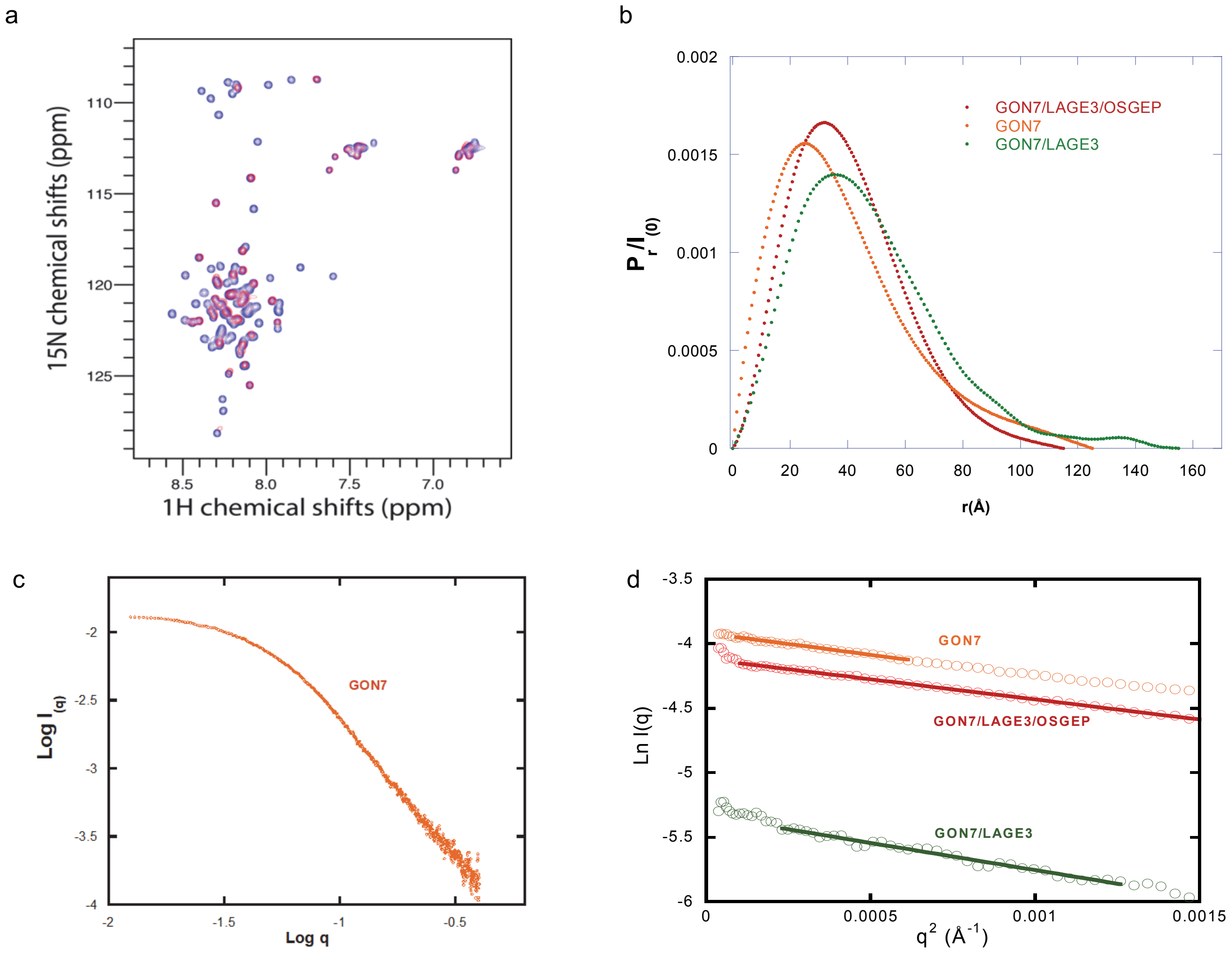

Supplementary Figure 6 | Characterization in solution of GON7 and GON7 complexes. (**a**) Overlap of 2D ^{15}N SOFAST-HMQC spectra[1] collected on 200 µM ^{15}N-labeled GON7 alone (blue spectrum) or 65 µM ^{15}N-labeled GON7-unlabeled LAGE3 (red spectrum) in a buffer composed of 20mM MES pH 6.5 200mM NaCl 5mM mercapto-ethanol in 90%/10% H2O/D2O. The NMR spectra were recorded at 20 °C on a Bruker Avance III 800 MHz equipped with a TCI cryoprobe. The expression of ^{15}N-labeled GON7 was achieved in the *E. coli* Gold strain in minimum media M68^{15}N at 37°C during 4 hours. The expression of LAGE3 was achieved in the *E.coli* Gold strain in rich medium at 37°C during 3 hours. Cells were then harvested by centrifugation, suspended in 35 mL of buffer A. GON7- ^{15}N and LAGE3 were purify independently as previously cited. 232 mM of LAGE3 were incubated with 156 mM of GON7-^{15}N on ice. The subunits or binary complex were purified by size exclusion chromatography (Superdex 200 10-300). The purest fractions (analyzed by SDS PAGE) were pooled and concentrated up to 200 µM and then analyzed by NMR. (**b**) Distance distribution (Pr) curves for GON7 (orange) and for the GON7/LAGE3 (green) and GON7/LAGE3/OSGEP (red) sub-complexes. The normalized distance distribution was calculated by BioXTAS RAW software that uses the Bayesian method for determining the inverse Fourier transform (IFT) of a scattering profile[2].(**c**) Log/log plot of the SAXS data for GON7. (**d**) Guinier plot of GON7 (orange), GON7/LAGE3 (green) and GON7/LAGE3/OSGEP (red).

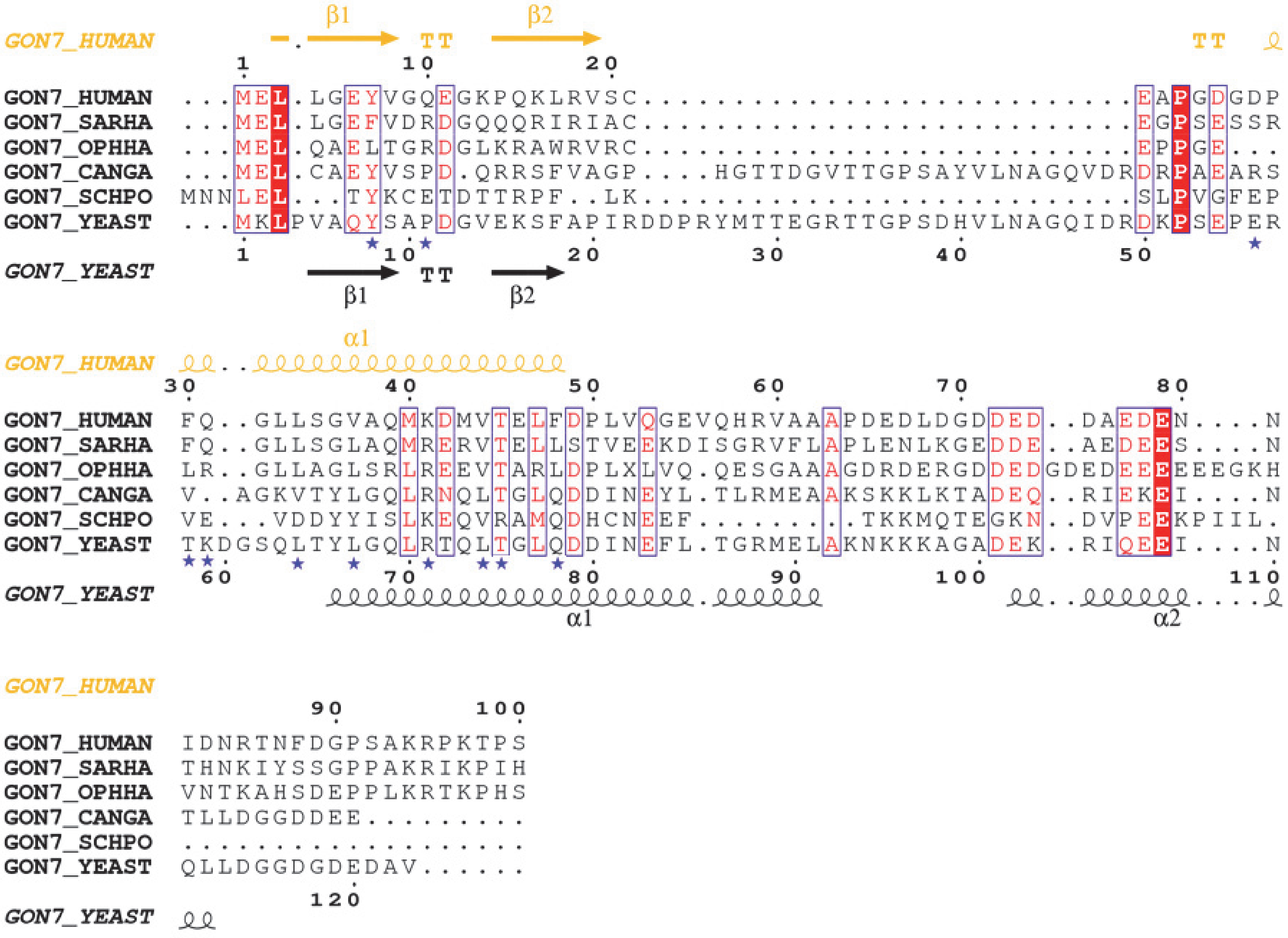

Supplementary Figure 7 | Structure based sequence alignment between human GON7 and yeast Gon7. Secondary structure elements were extracted from the crystal structures of GON7/LAGE3/OSGEP (top) and Gon7/Pcc1/Kae1 (bottom). Group 1 contains sequences from metazoans (*Homo sapiens*, *Sarcophilus harrisii* and *Ophiophagus hannah)* while group 2 contains sequences from fungi (*Candida glabrata, Schizosaccharomyces pombe and Saccharomyces cerevisiae*). Residues in contact with LAGE3 are marked by blue stars below the sequence.

a b

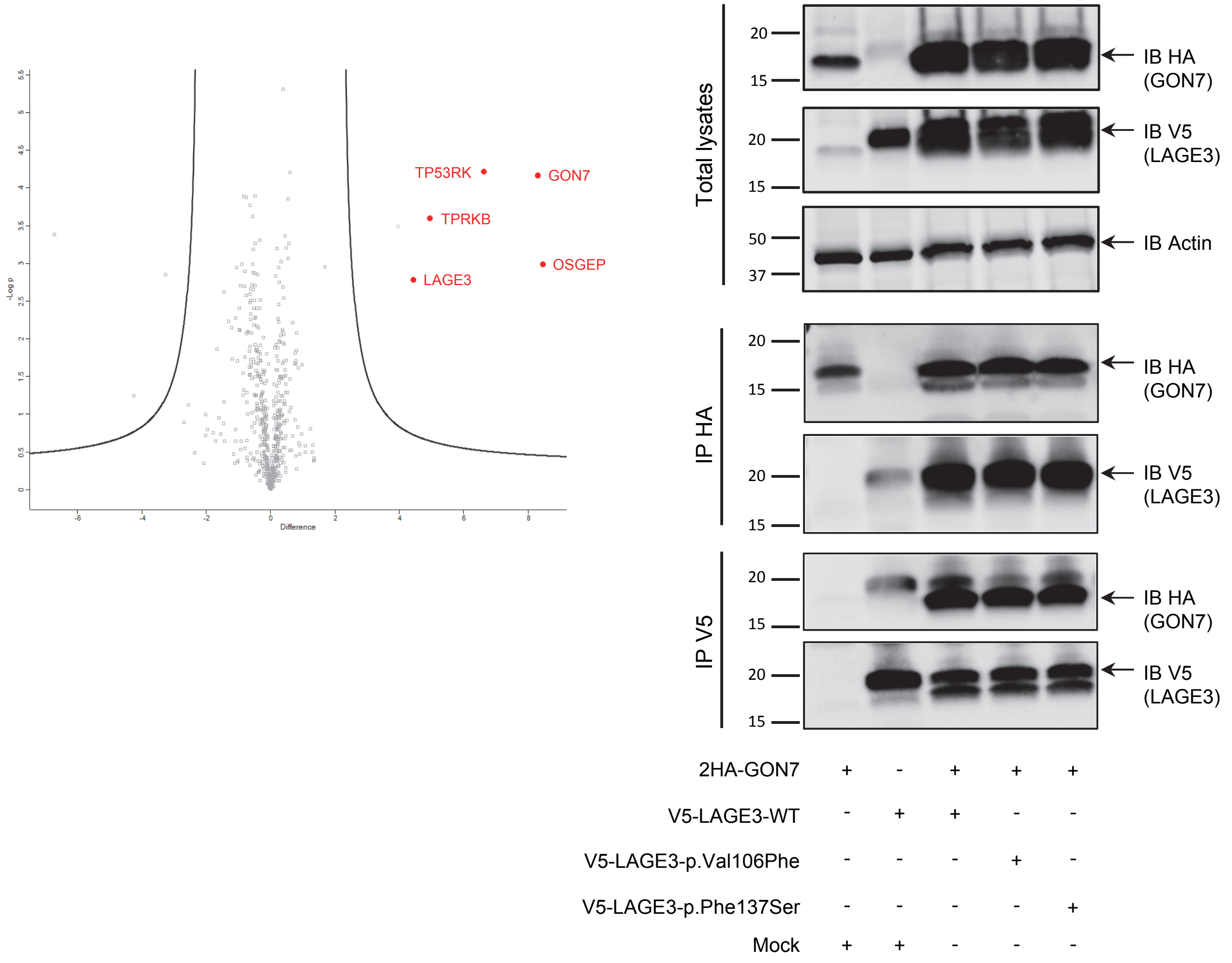

Supplementary Figure 8 | GON7 interactions with KEOPS complex subunits in human podocytes and with LAGE3 mutants. (**a**) Volcano plot representation of GON7-interacting proteins identified by mass spectrometry (nanoRSLC-Q Exactive PLUS MS) in 3 independent HA-immunoprecipitation experiments in a human podocyte cell line (empty HA-plasmid was used as control). X-axis reports the difference of the average of the logarithm of Label Free Quantification (LQF) intensities between the IP and its control; y-axis reports the negative logarithmic of t-test p value. Proteins showing a significant interaction are depicted outside of the black lines according to significance analysis (FRD=0.01, S0=2). Red dots indicate the four other human KEOPS subunits. (**b**) Lysates from HEK293T cells co-expressing HA-tagged GON7 and V5-tagged wild-type (WT) or mutant forms of LAGE3 were immunoprecipitated either with an anti-HA or anti-V5 antibody. Co-immunoprecipitated proteins were visualized by immunoblot (IB) using HA and V5 antibodies. Mock corresponds to empty vectors. Actin was used as a loading control.

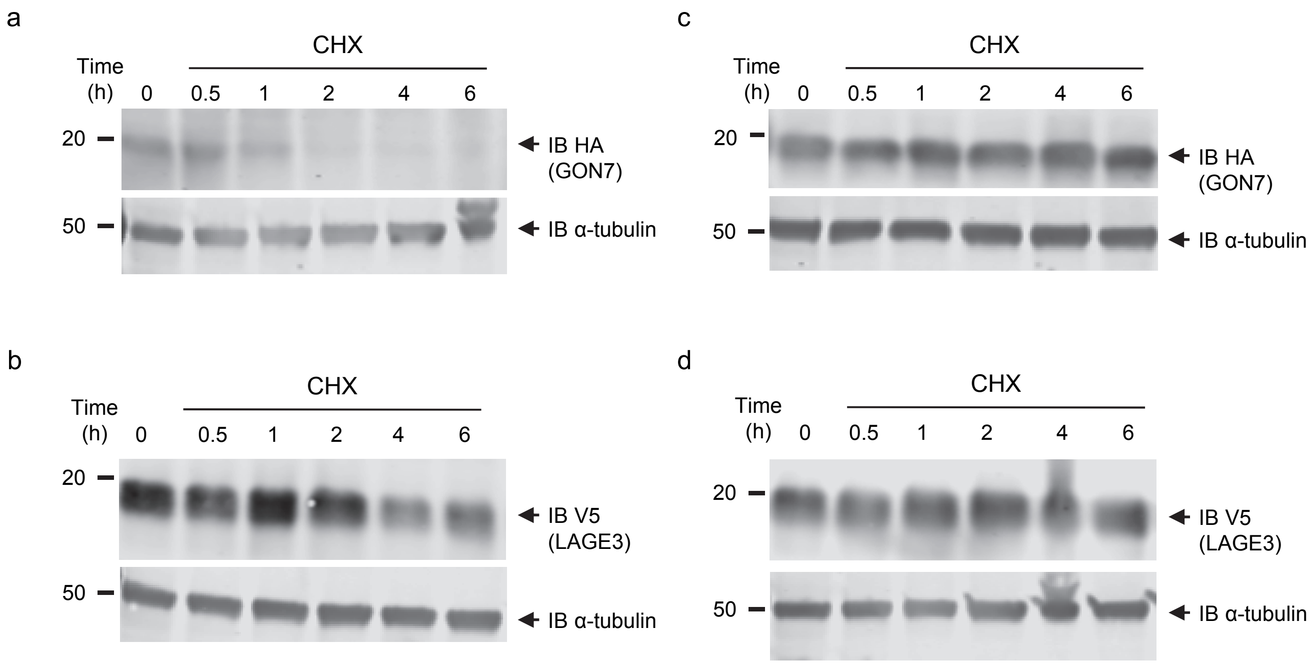

Supplementary Figure 9 | Cycloheximide chase experiments. HEK293T expressing either 2HA-tagged GON7 (**a**) or V5-tagged LAGE3 (**b**) alone or co-expressing both proteins (**c** and **d**) and subjected to treatment with cycloheximide to observe protein degradation (100 μM for 30min, 1 hour, 2 hrs, 4 hrs and 6 hrs) followed by western blotting of the cell lysates for both proteins with anti-HA (a and c) and anti-V5 (b and d) antibodies (experiments were performed 3 times).

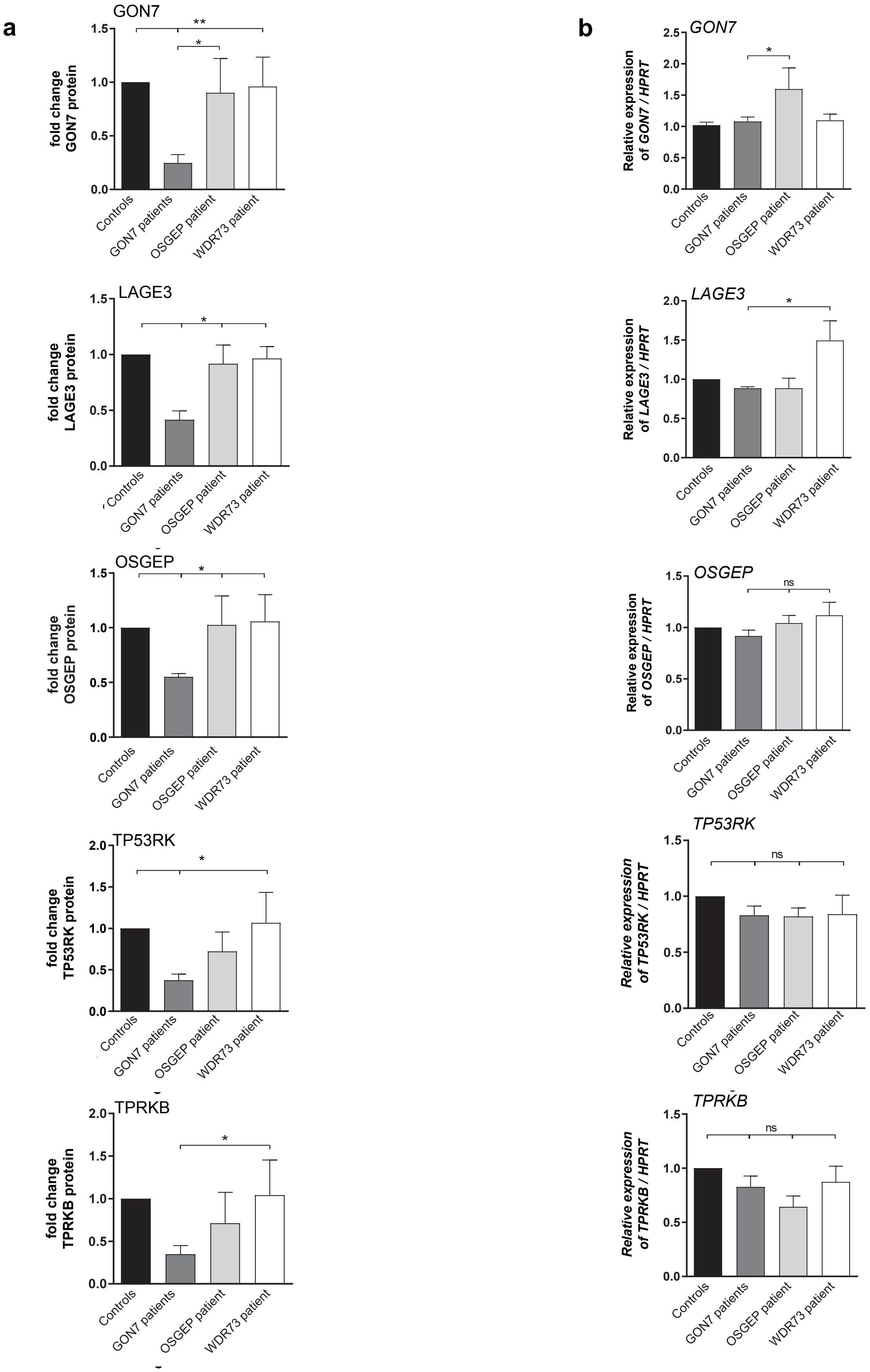

Supplementary Figure 10 | KEOPS subunits protein and mRNA expression levels in cells from GON7 affected individuals. Quantification of protein expression levels in (**a**) and transcript levels in (**b**) of the 5 KEOPS complex subunits. Proteins and mRNA were extracted from cell samples used in Figure 6d (mean ± s.e.m of *n*=3 experiments, with each experiment performed in triplicate; one-way ANOVA, Dunnett's *post-hoc* test).

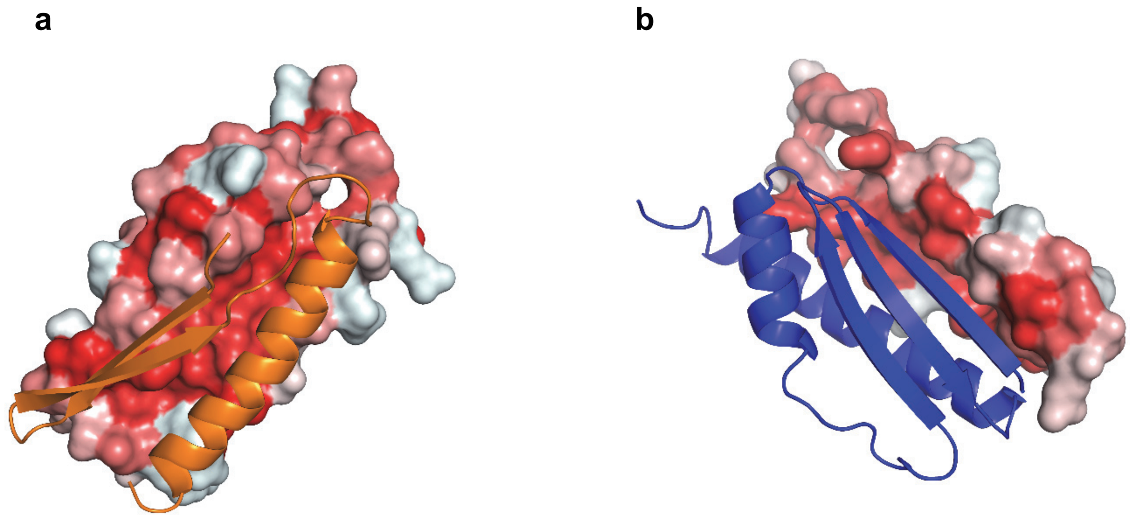

Supplementary Figure 11 | Hydrophobic surfaces at the GON7/LAGE3 interface. (**a**) Surface presentation of LAGE3, highlighting the hydrophobicity (increasing from white to red); GON7 presented in gold cartoon. (**b**) Surface presentation of GON7, highlighting the hydrophobicity (increasing from white to red); LAGE3 presented in blue cartoon.

a

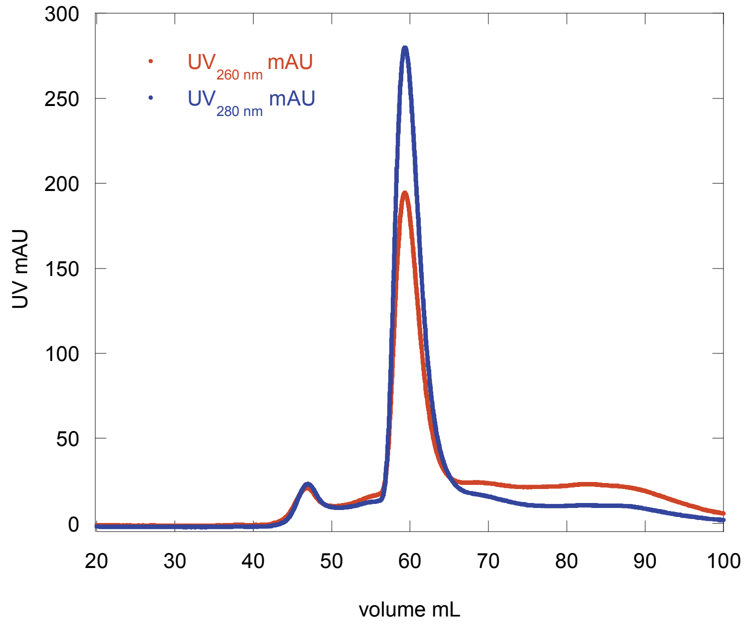

b

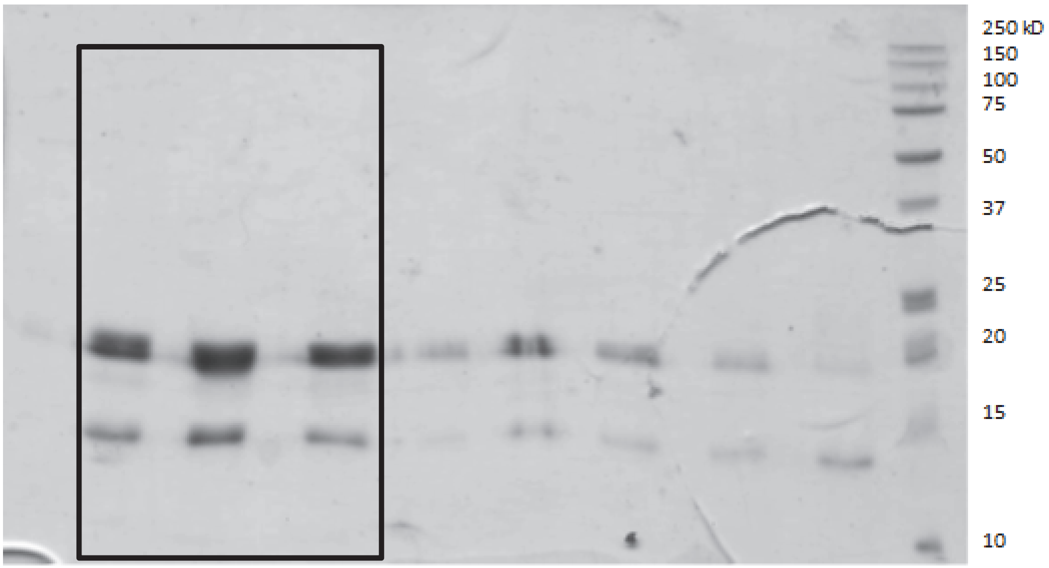

Supplementary Figure 12 | Gel filtration analysis of GON7/LAGE3 complex. (**a**) Gel filtration profile. (**b**) SDS-PAGE analysis of eluted fractions (black square: major peak fractions eluted at 59 mL)

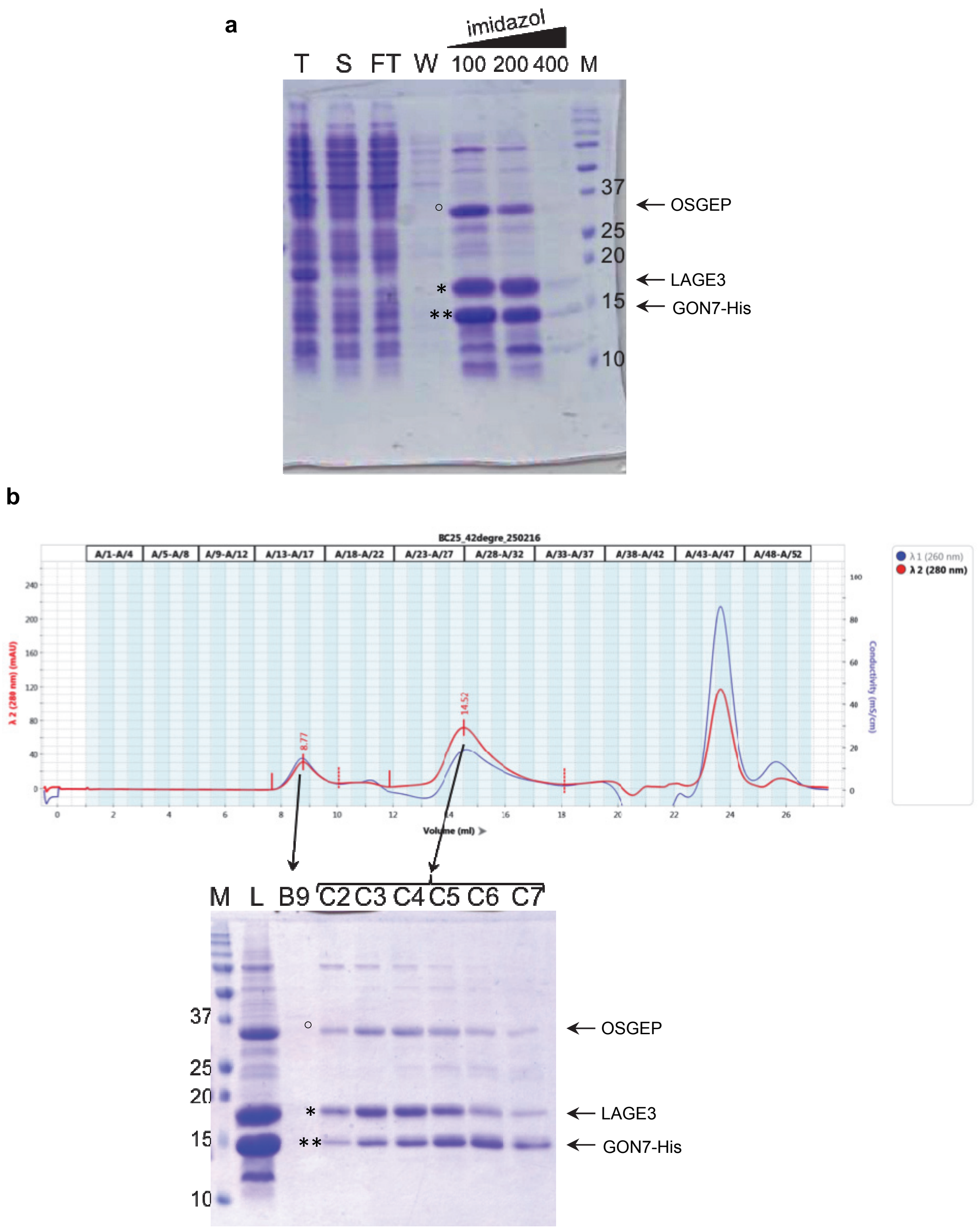

Supplementary Figure 13 | Co-purification of OSGEP/LAGE3/GON7-His subcomplex. (**a**) SDS-PAGE analysis of Ni^{2+} chromatography fractions. T: Total whole cell extract; S: soluble cell extract; FT: Flowthrough fraction; W: Wash fraction; 100, 200, and 400: elution fraction with 100 mM, 200 mM and 400 mM imidazol, respectively. (**b**) Analytical S200 size-exclusion chromatography of the 100 mM and 200 mM imidazole fractions obtained in (a). Below chromatogram is the SDS-PAGE analysis of the 2 peaks eluted at 8.77 mL (B9) and 14,52 mL (fractions C2 to C7). Numbers: molecular weight markers in kDa. (°): OSGEP, (*): LAGE3 and (**): GON7-His.

a b c d e

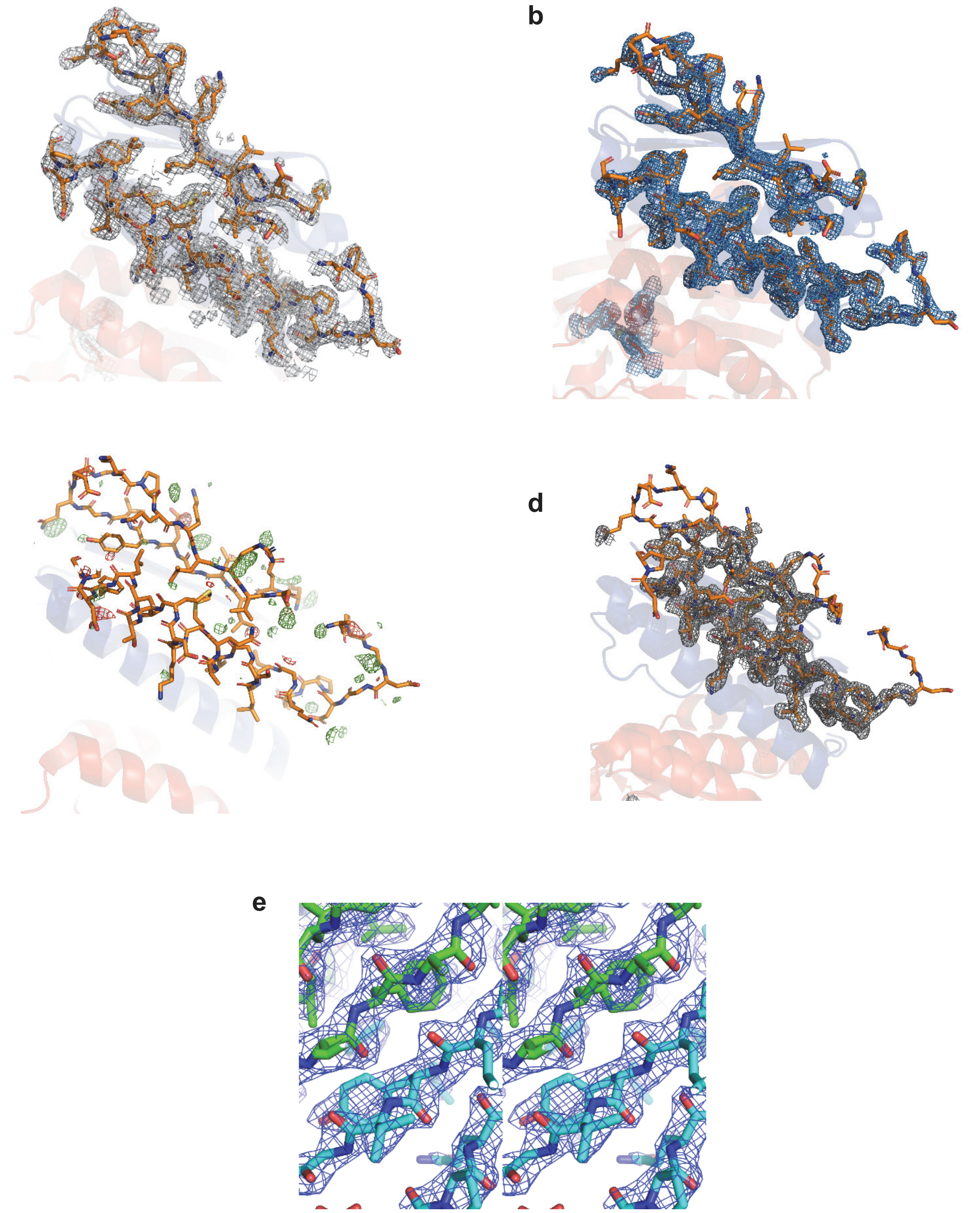

Supplementary Figure 14 | Calculated maps obtained from X-ray data. (**a**) A composite-omit map calculated by omitting 5% at a time showed good agreement with the unrefined structure indicating the phases were not dominated by model bias. (**b**) the $2mF_{obs}-DF_{calc}$ (contoured at 1σ) map covers protein residues 2-50 of GON7. (**c**) $mF_{obs}-DF_{calc}$ difference map contoured at +3.0σ (green) and −3.0σ (red) for the same protein region. (**d**) Prime and switch map. (**e**) 2Fo-Fc map of a few residues at the LAGE3 (green sticks) - GON7 (blue sticks) interface contoured at 1 sigma (stereo view).

The map (**b**) shows well-defined density around the vast majority of side chains and the difference map (**c**) shows no large discrepancies between the observed data (F_{obs}) and the model (F_{calc}).

Family -Individual	Nucleotide change	Amino acid change	Exon (Zygosity, Segregation)	gnomAD (AC/Hom/AN)	Gender	Ethnic origin	Parental Consan-guinity	Age of proteinuria (Age of ESRD) *Age at Death*	Extrarenal Manifestations	Biopsy (at age)	Therapy and response	Comments
GON7 **(5 families, NM_032490.4)**												
Family A<br>II-3<br>(N1560)	c.21 C>A	p.Tyr7*	Exon 1 (Hom), m(het), p(het)	1/0/249,098	M	Algeria	Yes	2 mon<br>(6 yrs)<br>33 yrs	Brain: Secondary microcephaly, developmental delay, choreoathetosic encephalopathy.<br>Cranial imaging (12 yrs) moderate but progressive cerebellar atrophy, cortical atrophy, ventricular dilation, thin corpus callosum.<br>Facies: dysmorphy: narrow forehead, large low-set ears, low nasal bridge, pinched nose, almond shaped eyes, hypotelorism, coarse hair.<br>Skeletal: abnormal skull shape.<br>Other: hiatal hernia with gastro-oesophageal reflux	FSGS (5 yrs)	SRNS	One affected sister died at 3 yrs of age. No available DNA.
II-4<br>(N1561)	c.21 C>A	p.Tyr7*	Exon 1 (Hom), m(het), p(het)	1/0/249,098	F	Algeria	Yes	4 mon<br>(4 yrs)<br>Alive with a functioning graft at 23 yrs	Brain: Secondary microcephaly, developmental delay.<br>Cranial imaging: moderate cerebral and cerebellar atrophy, thin corpus callosum.<br>Facies: narrow forehead, large low-set ears, low nasal bridge, pinched nose, almond shaped eyes, hypotelorism, coarse hair, hypertelorism.<br>Skeletal: abnormal skull shape mammary.<br>Other: hiatal hernia with gastro-oesophageal reflux	ND	No IS used	
II-6<br>(N1562)	c.21 C>A	p.Tyr7*	Exon 1 (Hom), m(het), p(het)	1/0/249,098	M	Algeria	Yes	5 mon<br>(6 yrs)<br>Alive with a functioning graft at 19 yrs	Brain: Secondary microcephaly, developmental delay.<br>Cranial imaging: moderate cerebral and cerebellar atrophy, myelination delay.<br>Facies: narrow forehead, large low-set ears, low nasal bridge, pinched nose, almond shaped eyes, hypotelorism.<br>Other: hiatal hernia with gastro-oesophageal reflux	ND	No IS used	
Family B<br>II-1<br>(N969)	c.21 C>A	p.Tyr7*	Exon 1 (Hom), m(het), p(het)	1/0/249,098	M	Algeria	No but same region	21 mon<br>(32 mon)<br>15 yrs	Brain: Secondary microcephaly, developmental delay.<br>Cranial imaging (2 yrs): moderate cerebral and cerebellar atrophy, atrophic corpus callosum, myelination delay.	DMS	SRNS	
II-3<br>(N768)	c.21 C>A	p.Tyr7*	Exon 1 (Hom), m(het), p(het)	1/0/249,098	F	Algeria	No but same region	12 mon<br>(33 mon)<br>3 yrs	Brain: Secondary microcephaly, developmental delay.<br>Cranial imaging (2 yrs): moderate cerebral and cerebellar atrophy, dilated lateral ventricles, myelination delay.	DMS	SRNS Ciclo-resistant NS	
II-4<br>(N1552)	c.21 C>A	p.Tyr7*	Exon 1 (Hom), m(het), p(het)	1/0/249,098	F	Algeria	No but same region	24 mon<br>(30 mon)<br>Alive with a functioning graft at 15 yrs	Brain: Secondary microcephaly, developmental delay.<br>Cranial imaging<br>- 3 yrs: cerebral and cerebellar atrophy, myelination delay.<br>- 5 yrs: stable cerebellar and cortical atrophy, ventricular dilation, thin corpus callosum, abnormal myelination.	DMS	No Is used	
Family C<br>II-1<br>(N3290)	c.21 C>A	p.Tyr7*	Exon 1 (Hom), m(het), p(het)	1/0/249,098	M	Algeria	yes	12 mon<br>(N/A)<br>Alive with normal renal function and heavy Pu at 9 yrs	Brain: Secondary microcephaly, developmental delay.<br>Cranial imaging (6 mon): moderate cerebral atrophy.	ND	No IS used	
II-2<br>(N2516)	c.21 C>A	p.Tyr7*	Exon 1 (Hom), m(het), p(het)	1/0/249,098	F	Algeria	yes	12 mon<br>(6 yrs)<br>Alive with a functioning graft at 15 yrs	Brain: Secondary microcephaly, developmental delay.<br>Cranial imaging (6 yrs): severe cerebellar atrophy, cortical atrophy, ventricular dilation, thin corpus callosum.<br>Other: sensorineural hearing loss	FSGS (18 mon)	SRNS Ciclo-resistant and mycophenolate mofetil-resistant NS	

II-3<br>(N3231)	c.21 C>A	p.Tyr7*	Exon 1 (Hom),<br>m(het), p(het)	1/0/249,098	F	Algeria	yes	8 mon<br>(N/A)<br>Alive with normal renal function and mild Pu at 7.5 yrs	Brain: Secondary microcephaly, developmental delay.<br>Cranial imaging: ND	ND	No IS used<br>ACE inhibitors and ARBs	
Family D<br>II-1<br>(N3780)	c.21 C>A	p.Tyr7*	Exon 1 (Hom),<br>m(het), p(het)	1/0/249,098	M	Algeria	yes	5 yrs<br>(N/A)<br>Alive with normal renal function and heavy Pu at 7.5 yrs	Brain: Secondary microcephaly, developmental delay no visual contact.<br>Cranial imaging: ND	ND	No IS used	An affected brother died when 5 years old, 3 mon after SRNS diagnosis and 2 mon after dialysis start
Family E<br>II-3<br>(DM)	c.19dup	p.Tyr7Leufs*16	Exon 1(Hom);<br>m(het), p(het);	0	M	Arab	yes	2 yrs<br>(3 yrs)<br>Alive with a functioning graft at 6 yrs	Brain: Microcephaly, neurodevelopmental delay<br>Cranial imaging (16 mon): cerebellar hypoplasia, cortical atrophy, ventricular dilation, thin corpus callosum, myelination delay.<br>Facies: facial dysmorphy	FSGS	SRNS	health sibling 1 (het); health sibling 2 (WT);
YRDC (2 families, NM_024640.3)												
Family F<br>II-1<br>(N4261)	c.251 C>T	p.Ala84Val §	Exon 1 (het) #	0	F	European	No	2 mon<br>(11 mon)<br>15 mon	Brain: hypotonia (4 mon), secondary microcephaly (6 mon), myoclonia,<br>Cranial imaging:<br>- 5 mon: normal<br>- 11 mon: progressive major cerebellar and cortical atrophy and very marked myelination defects.<br>Facies: facial dysmorphy.<br>Other: congenital hypothyroidism	DMS<br>(11 mon)	SRNS<br>Ciclo-resistant	
	c.721_724 del	p.Val241Ilefs*72	Exon 4 (Het) #	1/0/251,484								
Family G<br>II-1<br>(NG_010_02)	c.794_796 del	p.Leu265del	Exon 4 (Hom),<br>m(het), p(het)	0	M	European	Yes	1 mon<br>(1.5 mon)<br>1.5 mon	Brain: primary microcephaly.<br>Cranial imaging: ND<br>Skeletal: arachnodactyly.<br>Facies: facial dysmorphy.<br>Other: congenital hypothyroidism.	DMS and FPE<br>(1.5 mon)	No Is used	
II-2<br>(NG_010_01)	c.794_796 del	p.Leu265del	Exon 4 (Hom),<br>m(het), p(het)	0	F	European	Yes	Birth<br>(3 mon)<br>3 mon	Brain: primary microcephaly.<br>Cranial imaging (1 mon) : frontal simplified gyral pattern, left thalamic infarction, myelination delay.<br>Facies: facial dysmorphy.<br>Skeletal: arachnodactyly.<br>Other: congenital hypothyroidism.	ND	No Is used	

Supplementary Table 1. Mutations in *GON7* and *YRDC* genes in 14 individuals of 7 families with Galloway-Mowat syndrome.
ESRD, end-stage renal disease; FSGS, focal segmental glomerulosclerosis; DMS, diffuse mesangial sclerosis; FPE, foot process effacement; SRNS, steroid resistant nephrotic syndrome; NS, nephrotic syndrome; IS, immunosuppression; ACE, angiotensin converting enzyme; ARBs, Angiotensin receptor blockers, Pu, proteinuria; yrs, years; mon, months; AC, allele count; AN, allele number; Hom, homozygous; het, heterozygous; WT, wild-type; p, paternal; m, maternal; N/A, not applicable; ND, not done. **§** PolyPhen2 (0.709), SIFT (0.03) and Mutation taster (disease causing: 1) scores. **#** Each mutation has been inherited from a different parent. In addition, none of the variants described in our study was present in the homozygous or compound heterozygous state in our in-house database (n>16,000 individuals both from European and Northern African ancestry).

Variation	Gene	Min Pop Freq	Max Pop Freq	Family A		Family B		Family C		Family D
				N1560	N1561	N768	N969	N3231	N3290	N3780
14_92920423_C_A (rs17783624)	SLC24A4	AFR: 0.0444	ASJ: 0.26	H 0/59	H 0/33	h 15/13	H 0/62	H 0/138	H 0/121	H 0/85
14_92922681_A_G (rs7156906)	SLC24A4	EAS: 0.26	AFR: 0.48	H 0/16	H 0/15	h 3/11	H 1/20	H 0/48	H 0/53	H 0/44
14_92922713_T_C (rs7158400)	SLC24A4	AMR: 0.63	AFR: 0.89	H 0/27	H 0/29	H 0/24	H 0/39	H 0/88	H 0/102	H 0/86
14_92922900_G_A (rs77671856)	SLC24A4	EAS: 0.0824	AMR: 0.28	H 0/43	H 1/37	h 17/13	H 0/54	H 0/152	H 0/118	H 0/134
14_92959216_G_A (rs11623883)	SLC24A4	EAS: 0.28	FIN: 0.57	H 0/77	H 0/68	h 21/17	H 0/82	H 0/72	H 1/62	H 0/60
14_92960025_T_C (rs4904942)	SLC24A4	OTH: 1.00	OTH: 1.00	H 0/81	H 0/92	H 0/67	H 0/87	H 0/49	H 0/67	H 0/57
14_92960172_G_A (rs12888354)	SLC24A4	EAS: 0.30	FIN: 0.56	H 0/44	H 0/53	h 17/16	H 0/38	H 0/7	H 0/11	H 0/15
14_92980553_C_A (rs71430764)	RIN3	EAS: 0.00000	NFE: 0.0577	H 0/22	H 0/14	h 5/5	H 0/14	H 0/40	H 0/32	H 0/21
14_93118669_G_A (rs3742716)	RIN3	AFR: 0.0644	EAS: 0.44	H 0/130	H 0/123	H 0/81	H 1/101	H 0/229	H 3/224	H 1/222
14_93154538_TGGC_T (rs570458246)	RIN3	AMR: 0.54	EAS: 0.79	H 0/133	H 0/98	H 0/65	H 0/104	H 2/216	H 1/252	H 0/248
14_93178191_A_G (rs1951708)	LGMN	AFR: 0.99	OTH: 1.00	H 0/221	H 0/253	H 0/167	H 0/257	H 0/498	H 0/435	H 0/495
14_93198972_A_T (rs12885208)	LGMN	EAS: 0.0300	FIN: 0.26	H 0/66	H 0/89	H 0/60	H 0/81	H 0/73	H 1/54	H 0/67
14_93199203_T_A (rs1613441)	LGMN	ASJ: 0.62	AFR: 0.93	H 0/248	H 0/257	H 0/153	H 0/248	H 0/107	H 0/106	H 0/111
14_93404555_A_G (rs4638507)	ITPK1	ASJ: 0.93	EAS: 1.00	H 0/84	H 0/98	H 0/61	H 1/75	H 0/9	H 0/3	H 0/7
14_93407301_C_A (rs13316)	ITPK1	AFR: 0.35	ASJ: 0.47	H 0/31	H 1/31	H 0/22	H 0/25	H 0/41	H 0/41	H 0/46
14_93412609_A_G (rs1998054)	ITPK1	ASJ: 0.33	EAS: 0.78	H 0/24	H 0/24	H 0/15	H 0/32	H 1/60	H 0/54	H 0/71
14_93412743_G_A (rs2295394)	ITPK1	ASJ: 0.0348	EAS: 0.22	H 0/64	H 0/70	H 0/32	H 0/66	H 0/202	H 0/168	H 0/153
14_93418155_T_C (rs2273392)	ITPK1	ASJ: 0.0497	EAS: 0.22	H 0/12	H 0/20	H 0/10	H 0/12	H 0/33	H 0/32	H 0/35
14_93418156_G_C (rs2273391)	ITPK1	ASJ: 0.0497	EAS: 0.22	H 0/12	H 0/20	H 0/10	H 0/12	H 0/34	H 0/33	H 0/35
14_93428802_T_A (rs2295393)	ITPK1	AFR: 0.0462	EAS: 0.17	H 0/64	H 0/54	H 0/36	H 0/60	H 2/213	H 1/147	H 0/182
14_93649501_A_G (rs1046099)	MOAP1	ASJ: 0.61	AFR: 0.75	H 0/37	H 0/44	H 0/32	H 0/41	H 0/33	H 0/27	H 0/38
14_93651992_T_C (rs1268371)	TMEM251	EAS: 0.0728	AMR: 0.19	H 0/18	H 0/7	H 0/8	H 0/13	H 0/39	H 0/45	H 0/35
14_93673362_G_T	GON7	OTH: 0.00000	NFE: 0.00001	H 0/69	H 0/52	H 0/36	H 0/73	H 0/51	H 0/50	H 0/44
14_93693422_C_T (rs2905)	UBR7	AFR: 0.062	ASJ: 0.34	H 0/270	H 0/202	H 1/181	H 1/244	H 0/409	H 0/391	H 0/388
14_93693604_T_G (rs10144530)	UBR7	SAS: 0.31	FIN: 0.53	H 0/61	H 0/44	H 0/44	H 0/69	H 0/29	H 0/17	H 0/20
14_93712645_TAA_T (rs566777055)	BTBD7	AMR: 0.26	AFR: 0.44	H 2/37	H 1/29	H 0/23	H 1/26	H 8/74 90%	H 3/86	H 3/55
14_93720181_T_C (rs8012209)	BTBD7	FIN: 0.31	AFR: 0.60	H 0/31	H 0/42	H 0/25	H 0/43	H 1/71	H 0/106	H 0/97
14_93762162_C_T (rs2273639)	BTBD7	FIN: 0.29	AFR: 0.53	H 0/10	H 0/18	H 0/16	H 0/16	H 0/55	H 0/42	H 0/41
14_93799221_G_A (rs2273640)	BTBD7	AFR: 0.09	EAS: 0.35	H 0/49	H 0/35	H 0/24	H 2/54	H 0/49	H 0/75	H 0/66
14_93799285_ACCG_A (rs146686071)	BTBD7	FIN: 0.47	AFR: 0.88	H 0/14	H 0/15	H 0/12	H 1/21	H 0/13	H 0/16	H 0/15
14_93943998_G_A (rs61992606)	UNC79	AFR: 0.27	EAS: 0.65	H 0/242	H 2/352	H 0/206	H 1/356	H 0/301	H 0/287	H 0/271
14_93953908_A_AT (rs33942732)	UNC79	AFR: 0.72	EAS: 0.93	H 1/13	H 1/32	H 0/22	H 0/18	H 1/64	H 2/67	H 4/80
14_93954192_C_T (rs12884458)	UNC79	AFR: 0.25	EAS: 0.64	H 0/23	H 0/36	H 0/21	H 0/35	H 1/112	H 0/102	H 0/84
14_93962691_G_A (rs8006093)	UNC79	EAS: 0.93	AFR: 1.00	H 0/4	H 0/11	H 0/4	H 0/8	H 0/35	H 0/57	H 0/23
14_93998806_C_A (rs942068)	UNC79	AFR: 0.79	EAS: 0.93	H 0/16	H 0/29	H 0/12	H 0/15	H 1/36	H 0/99	H 0/47
14_94008753_A_G (rs12893237)	UNC79	AFR: 0.81	EAS: 0.93	H 0/24	H 0/44	H 0/16	H 0/26	H 0/122	H 0/98	H 0/124
14_94391699_G_A (rs10141024)	FAM181A	AMR: 0.38	EAS: 0.62	H 0/79	H 0/71	H 0/60	H 2/70	H 0/205	H 0/163	H 0/180
14_94394039_G_GT (rs573718885)	FAM181A	EAS: 0.000	NFE: 0.012	H 0/15	H 0/8	H 0/6	H 0/9	H 0/46	H 0/36	H 0/24
14_94400431_T_C (rs11625548)	ASB2	EAS: 0.28	ASJ: 0.69	H 0/39	H 0/43	H 0/28	H 0/59	H 0/25	H 0/30	H 0/35
14_94405871_G_C (rs10873442)	ASB2	AMR: 0.62	ASJ: 0.92	H 2/96	H 0/75	H 1/79	H 0/98	H 0/229	H 0/243	H 0/214
14_94406096_A_C (rs7155320)	ASB2	AMR: 0.69	ASJ: 0.93	H 0/8	H 0/10	H 0/4	H 0/8	H 0/25	H 0/18	H 0/8
14_94410447_T_C (rs10134105)	ASB2	AMR: 0.70	ASJ: 0.93	H 0/121	H 0/118	H 0/94	H 0/108	H 0/41	H 0/53	H 0/48
14_94417421_G_A (rs7147919)	ASB2	AFR: 0.37	EAS: 0.79	H 0/134	H 0/121	H 0/83	H 0/114	H 0/309	H 0/276	H 0/298
14_94417541_A_G (rs4277287)	ASB2	AMR: 0.63	ASJ: 0.93	H 0/37	H 0/39	H 0/25	H 0/48	H 0/122	H 0/118	H 0/122
14_94417586_G_A (rs4483793)	ASB2	AFR: 0.45	EAS: 0.79	H 0/16	H 0/18	H 0/12	H 0/18	H 0/53	H 0/62	H 0/57
14_94417675_A_C (rs4433745)	ASB2	AFR: 0.40	EAS: 0.77	H 0/7	H 0/4	H 0/3	H 0/8	H 0/9	H 0/19	H 0/11
14_94419888_A_G (rs4243701)	ASB2	AMR: 0.76	EAS: 1.00	H 0/19	H 0/18	H 0/7	H 0/8	H 0/32	H 0/41	H 0/19
14_94594768_T_G (rs958187)	IFI27L2	EAS: 0.56	AFR: 0.94	H 0/66	H 0/51	H 0/41	H 0/45	H 0/177	H 0/137	H 0/158
14_94700138_C_G (rs4905172)	PPP4R4	FIN: 0.94	SAS: 1.00	H 0/39	H 0/55	H 0/27	H 0/50	H 0/119	H 0/149	H 0/105
14_94776401_T_C (rs11160168)	SERPINA6	ASJ: 0.57	EAS: 0.99	H 0/29	H 0/29	H 0/24	h 10/10	H 0/62	H 0/75	H 1/87
14_94833033_T_C (rs1956172)	SERPINA2P	AFR: 0.53	EAS: 0.98	H 0/138	H 0/125	H 0/84	H 0/130	H 0/272	H 0/267	H 0/262
14_94909630_C_T (rs3818257)	SERPINA11	EAS: 0.61	FIN: 0.86	H 0/65	H 0/77	H 0/47	h 37/29	H 0/210	H 0/199	H 0/194
14_94914455_A_T (rs10129672)	SERPINA11	EAS: 0.61	FIN: 0.82	H 0/16	H 0/22	H 0/11	H 9/3	H 0/46	H 0/60	H 0/48
14_94929541_G_A (rs2224418)	SERPINA9	AFR: 0.44	SAS: 0.86	H 0/100	H 0/105	H 0/56	h 54/47	H 0/211	H 0/245	H 1/218
14_94931016_T_G (rs8020984)	SERPINA9	EAS: 0.63	SAS: 0.86	H 0/109	H 0/128	H 0/57	h 76/57	H 0/211	H 0/193	H 0/181
14_94931105_A_G (rs11628722)	SERPINA9	AFR: 0.44	SAS: 0.86	H 0/146	H 1/206	H 0/75	h 86/86	H 0/418	H 0/436	H 0/419

Supplementary Table 2: Haplotype analysis in all index patients homozygous for *GON7* p.Tyr7* mutation. Exome sequencing data were used to show the genotypes of 55 SNP spanning 2 Mb in the region flanking the *GON7* locus. A common haplotype (in red) is present in the 7 patients tested : mimum size 1.58 Mb (rs3742716 to rs4905172), maximum size : 1.79 Mb (rs71430764 to rs11160168). Variation : Chromosome_Genomic position on GRCh37_Nucleotide change (SNP Reference) ; Min Pop Freq : Population with the lowest frequency in controls (Gnomad) ; Max Pop Freq : Population with the highest frequency in controls (Gnomad) ; AFR : African/African American, AMR : Latino, ASJ : Ashkenazi Jewish, EAS : East Asian, FIN : Finnish, NFE : Non-Finnish European, SAS : South Asian, OTH : Other ; H : Homozygous, h : heterozygous. Ratio number of wild type reads/number of of reads with the variant.

plasmid name	sequence
>BC47_YRDC-WT	CCATGGGTCATCACCATCACCATCACGAGAACCTGTACTTCCAGGGCCATATGCGCGCCGCGGTGGCGGCCAGCGTGGGCCTGAGCGAAGGTCCGGCCGGCAGCCGCAGCGGTCGCTTATTCCGCCCGCCGA<br>GCCCGGCCCCGGCGGCCCCGGGCGCCCGTCTGTTACGCCTGCCGGGTAGCGGCGCCGTGCAGGCCGCGAGCCCGGAACGCGCCGGCTGGACCGAGGCGCTGCGTGCCGCCGTGGCCGAACTGCGCGCCGGCG<br>CCGTGGTGGCCGTGCCGACCGATACGCTGTACGGCCTGGCCTGCGCGGCGAGCTGCTCGGCGGCCCTGCGCGCGGTGTACCGCCTGAAAGGTCGCAGCGAAGCCAAACCGCTGGCCGTTTGCTTAGGCCGCG<br>TGGCCGATGTCTACCGCTACTGCCGTGTGCGCGTGCCGGAGGGCTTACTGAAAGACTTACTGCCGGGTCCGGTGACCCTGGTGATGGAACGCAGCGAGGAACTGAACAAAGACCTGAACCCGTTTACGCCG<br>TTAGTGGGCATTCGTATTCCGGATCATGCCTTTATGCAAGACCTGGCGCAGATGTTTGAAGGTCCGTTAGCCCTGACCAGCGCCAACCTGAGCAGCCAGGCCAGCTCGCTGAATGTCGAAGAGTTCCAGGAT<br>CTGTGGCCGCAGTTAAGCCTGGTTATTGATGGTGGCCAGATTGGTGATGGCCAGAGCCCGGAATGTCGCCTGGGCAGCACCGTGGTTGATTTATCTGTGCCGGGTAAATTTGGCATCATTCGTCCGGGCTGC<br>GCCCTGGAAAGCACCACGGCCATCCTGCAACAGAAATACGGCCTGTTACCGAGCCATGCGAGCTATCTGTAAGGATCC
>BC48_YRDC-A84V	CCATGGGTCATCACCATCACCATCACGAGAACCTGTACTTCCAGGGCCATATGCGCGCCGCGGTGGCGGCCAGCGTGGGCCTGAGCGAAGGTCCGGCCGGCAGCCGCAGCGGTCGCTTATTCCGCCCGCCGA<br>GCCCGGCCCCGGCGGCCCCGGGCGCCCGTCTGTTACGCCTGCCGGGTAGCGGCGCCGTGCAGGCCGCGAGCCCGGAACGCGCCGGCTGGACCGAGGCGCTGCGTGCCGCCGTGGCCGAACTGCGCGCCGGCG<br>CCGTGGTGGTTGTGCCGACCGATACGCTGTACGGCCTGGCCTGCGCGGCGAGCTGCTCGGCGGCCCTGCGCGCGGTGTACCGCCTGAAAGGTCGCAGCGAAGCCAAACCGCTGGCCGTTTGCTTAGGCCGCG<br>TGGCCGATGTCTACCGCTACTGCCGTGTGCGCGTGCCGGAGGGCTTACTGAAAGACTTACTGCCGGGTCCGGTGACCCTGGTGATGGAACGCAGCGAGGAACTGAACAAAGACCTGAACCCGTTTACGCCG<br>TTAGTGGGCATTCGTATTCCGGATCATGCCTTTATGCAAGACCTGGCGCAGATGTTTGAAGGTCCGTTAGCCCTGACCAGCGCCAACCTGAGCAGCCAGGCCAGCTCGCTGAATGTCGAAGAGTTCCAGGAT<br>CTGTGGCCGCAGTTAAGCCTGGTTATTGATGGTGGCCAGATTGGTGATGGCCAGAGCCCGGAATGTCGCCTGGGCAGCACCGTGGTTGATTTATCTGTGCCGGGTAAATTTGGCATCATTCGTCCGGGCTGC<br>GCCCTGGAAAGCACCACGGCCATCCTGCAACAGAAATACGGCCTGTTACCGAGCCATGCGAGCTATCTGTAAGGATCC
>BC49_YRDC-del165	CCATGGGTCATCACCATCACCATCACGAGAACCTGTACTTCCAGGGCCATATGCGCGCCGCGGTGGCGGCCAGCGTGGGCCTGAGCGAAGGTCCGGCCGGCAGCCGCAGCGGTCGCTTATTCCGCCCGCCGA<br>GCCCGGCCCCGGCGGCCCCGGGCGCCCGTCTGTTACGCCTGCCGGGTAGCGGCGCCGTGCAGGCCGCGAGCCCGGAACGCGCCGGCTGGACCGAGGCGCTGCGTGCCGCCGTGGCCGAACTGCGCGCCGGCG<br>CCGTGGTGGCCGTGCCGACCGATACGCTGTACGGCCTGGCCTGCGCGGCGAGCTGCTCGGCGGCCCTGCGCGCGGTGTACCGCCTGAAAGGTCGCAGCGAAGCCAAACCGCTGGCCGTTTGCTTAGGCCGCG<br>TGGCCGATGTCTACCGCTACTGCCGTGTGCGCGTGCCGGAGGGCTTACTGAAAGACTTACTGCCGGGTCCGGTGACCCTGGTGATGGAACGCAGCGAGGAACTGAACAAAGACCTGAACCCGTTTACGCCG<br>TTAGTGGGCATTCGTATTCCGGATCATGCCTTTATGCAAGACCTGGCGCAGATGTTTGAAGGTCCGTTAGCCCTGACCAGCGCCAACCTGAGCAGCCAGGCCAGCTCGCTGAATGTCGAAGAGTTCCAGGAT<br>CTGTGGCCGCAGTTAAGCCTGGTTATTGATGGTGGCCAGATTGGTGATGGCCAGAGCCCGGAATGTCGCCTGGGCAGCACCGTGGTTGATTTATCTGTGCCGGGTAAATTTGGCATCATTCGTCCGGGCTGC<br>GCCCTGGAAAGCACCACGGCCATCCAACAGAAATACGGCCTGTTACCGAGCCATGCGAGCTATCTGTAAGGATCC

Supplementary Table 3 | Sequences for expression of YRDC-WT and mutants for NMR-1D spectra and enzymatic activity assays.

	GON7	GON7-LAGE3	GON7-LAGE3-OSGEP
**Data collection parameters**			
Instrument	SWING (SOLEIL)		
Detector	CCD-based AVIEX		
Beam geometry	0.8 mm x 0.15 mm		
Wavelength [Å]	1.0		
q-range [$Å^{-1}$]	$0.007 < q < 0.50$		
Absolute scaling	Comparison with scattering from pure H_2O		
Exposure time [s]	2		
Temperature [K]	283		
SEC-SAXS column	Biosec 3 Agilent		
Loading concentration [mg/mL]	6.7	0.7<br>4.2	1.48
Injection volume [µL]	65		
Solvent (solvent blanks taken from SEC flowthrough prior to elution of protein)	20 mM MES, 200 mM NaCl, 5 mM 2-mercaptoethanol		
Flow rate [$mL.min^{-1}$]	0.3		
Extinction coefficient [A_{280} 0.1% (w/v)]	0.238	0.344	0.563
Partial specific volume [$cm^3.g^{-1}$] [a]	0.7085	0.7155	0.7321
Average Concentration [$g.L^{-1}$] in combined data frames	~1.50	~0.10	~0.35
**Software used for SAXS data reduction and analysis**			
Foxtrot	Swing in-house software		
PRIMUS	ATSAS 2.8 suite, Franke et al. J. Appl. Cryst. 2017		
BUNCH	Petoukhov & Svergun, D.I. Biophys J.2005		
**Structural parameters**			
I(0) Guinier [cm^{-1}]	0.01988±0.00007	0.00483±0.00004	0.01625±0.00002
R_g Guinier [Å]	31.8±0.4	35.6±0.5	30.6±0.1
qRg-range	0.30-0.79	0.54-1.26	0.31-1.29
I(0) p(r) [cm^{-1}]	0.01988±0.00005	0.00493±0.00006	0.01634±0.00003
R_g p(r) [Å]	32.4±0.2	38.1±0.8	31.4±0.1
q-range [$Å^{-1}$]	0.0096 - 0.30	0.0152 - 0.40	0.01 - 0.40
D_{Max} [Å]	125	155	115
**Molecular mass determination**			
Mol . L -1$MM_{sequence}$ [kDa] [b]	12.59	29.05	62.88
$MM_{I(0)/c}$ [kDa]	14.7	56.5	58.5
$MM_{bayesian}$ [kDa] [c]<br>Credibility interval		65.45<br>63.1-75.3	59.5<br>57.5-64.5
**BUNCH analysis**			
Bunch Model number			10
q-range [$Å^{-1}$]			0.01 - 0.40
χ^2 (Crysol)			0.3
SAS BDB ID	SASDFK8	SASDFM8	SASDFL8

[a] Calculated from the sequence using the program sednterp.

[b] The calculated masses were derived from the sequences.

[c] Molecular mass M obtained using the bayesian inference approach which combines four concentration independent MM estimators[3]. This mass determination does not depend on the value of the protein concentration c and provides a useful complement to the derivation of M from the I(0)/c value . Nevertheless this method is not suitable for strongly unstructured protein due to the plateau of $I(q)q^2$ and the I(0)/c method must be preferred in the case of GON7.

Supplementary Table 4 | SAXS results and data collection parameters.

**Data collection**	
Wavelength (Å)	0.978570
Space group	$P4_3$
Cell (Å,°)	a=105.12 b=105.12 c= 51.35<br>α=β=γ= 90
Resolution	46.14 - 1.95 (2.06 – 1.95)
R meas (%)	7.1 (118.8)
CC ½ (%)	99.9 (58.5)
I/σ(I)	15.97 (1.38)
Completeness (%)	99.93 (99.88)
**Refinement statistics**	
Unique reflections	41188 (4071)
Rwork / Rfree	0.1809 / 0.2106
Total atoms: protein / solvent	3845 / 223
Average B-factors ($Å^2$)	54.83
RMSD bonds (Å)/ RMSD angles (°)	0.014/ 1.62
Ramachadran favored (%)	97.38
Ramachadran allowed (%)	2.62
Ramachadran disallowed (%)	0

Supplementary Table 5 | Crystallographic data collection and structure refinement statistics. Values in parentheses are for the highest-resolution shell.

Gene	Gene accession number	shRNA target sequence
GON7	NM_032490.5	CAAAGATAGTAAAGGCTTAGA
LAGE3	NM_006014.4	CTTTCTTGACCAGCTTTCCCT
OSGEP	NM_017807.3	GGATTAACCTCCCAGGATATC
YRDC	NM_024640.4	CAAGACTTGGCTCAGATGTTT

Supplementary Table 6 | shRNA targets for gene silencing

Gene	Forward primer	Reverse primer
GON7 qPCR	5'-CAGACGAGGACTTGGACGGTG-3'	5'-TTGGCCGTTTTGCAGATGGT-3'
LAGE3 qPCR	5'-GGGTGGTTGGGAAGGATCTC-3'	5'-GATGACGGAAATTCGGAGCA-3'
OSGEP qPCR	5'-GTGATTGCATACTCGGAACA-3'	5'-TGGACTTGGGTCGTTAGAAA-3'
YRDC qPCR	5'-GGGCTCCTGAAAGACCTACT-3'	5'-GGAATCCGAATGCCTACAAG-3'
TP53RK qPCR	5'-CTGGAATATCTGCCCCAGTTG-3'	5'-CACTGAGCCTTCAATTTCTTCCAT-3'
TPRKB qPCR	5'-CATGGAAGGCACCATCGAT-3'	5'-CTGCCACAAGTATCTGAAATGGA-3'
YRDC Exon 1	5'-GTCGTGGTCCCTTTAAGCTG-3'	5'-AAGCCTGTCACCGGAAAC-3'
YRDC Exon 2 & 3	5'-GATTCCTTTTCTACGTCTGCC-3'	5'-CATCCCAGTGTACTATCAATGC-3'
YRDC Exon 4 & 5	5'-GCCTTTGATGACTGCGT-3'	5'-ATAGTATCCAGCACCAGGTCTT-3'
GON7 Exon 1	5'-AGTGCCAGGAACCAATGACA-3'	5'-GGTCACTGCAGCACCGTCTC-3'
GON7 Exon 2	5'-GCAGGTTTTATTAATCCCTA-3'	5'-CTCTTTGACAAAATTGTCCC-3'

Supplementary Table 7 | sequence primers for qRT-PCR and genomic sequencing (exons)

Vector name	Sequence
pET21a-LAGE3_hisTEV_op<br>(cloned between *NdeI* and *EcoRI*)	CATATGCATCACCATCACCATCACGAAAACCTGTATTTTCAGGGCCGTGACGCGG<br>ACGCAGACGCAGGCGGTGGCGCTGACGGCGGGGACGGCCGCGGTGGCCACAGCTG<br>CCGCGGGGGCGTGGACACCGCCGCAGCTCCGGCCGGTGGCGCTCCGCCAGCGCAC<br>GCGCCGGGTCCGGGCCGTGACGCCGCGTCTGCGGCCCGCGGGTCACGTATGCGCC<br>CGCACATCTTCACCCTGAGCGTGCCATTCCCGACCCCATTGGAGGCGGAAATCGC<br>CCACGGTTCCCTGGCACCAGATGCCGAGCCGCACCAACGTGTGGTTGGGAAGGAT<br>CTGACCGTGAGTGGCCGTATCCTGGTCGTCCGCTGGAAAGCTGAAGACTGTCGCC<br>TGTTGCGTATTTCCGTCATCAACTTTCTTGACCAGCTTTCCCTGGTGGTGCGCAC<br>CATGCAGCGCTTTGGTCCGCCAGTTTCCCGCTAAGAATTC
pET24d-C14_hisTEV_op<br>(cloned between *NcoI* and *XhoI*)	CCATGGGGCATCACCATCACCATCACGAAAACCTGTATTTTCAGGGCGAGCTGCT<br>GGGTGAGTACGTCGGGCAGGAAGGGAAGCCGCAGAAGCTGCGTGTGTCCTGTGAG<br>GCGCCGGGTGACGGCGACCCATTCCAGGGCCTGTTGTCTGGCGTGGCCCAGATGA<br>AGGACATGGTAACGGAATTGTTCGACCCACTGGTACAGGGTGAAGTGCAGCACCG<br>CGTGGCGGCGGCTCCGGACGAGGACTTGGACGGTGATGATGAAGATGATGCAGAA<br>GATGAAAATAACATTGATAACCGTACTAACTTCGATGGTCCATCTGCAAAACGCC<br>CAAAAACCCCGTCTTAAGCTCGAG
pET24a-BC25_hKEOPS_opt<br>(cloned between *NdeI* and *XhoI*)	CATATGCCGGCGGTGCTGGGTTTTGAAGGCAGCGCCAACAAAATTGGCGTGGGCG<br>TGGTGCGCGATGGCAAAGTGCTGGCGAACCCGCGTCGCACCTATGTGACGCCGCC<br>GGGCACCGGCTTTCTGCCGGGTGATACCGCCCGCCATCACCGTGCGGTTATCCTG<br>GATCTGCTGCAGGAAGCACTGACGGAAAGCGGCTTAACCAGCCAGGATATCGACT<br>GCATTGCCTACACCAAAGGCCCGGGCATGGGTGCCCCGCTGGTTAGCGTGGCGGT<br>TGTGGCCCGTACCGTGGCCCAGCTGTGGAATAAACCGCTGGTGGGTGTGAACCAC<br>TGTATTGGCCACATTGAAATGGGCCGCCTGATCACCGGCGCCACCAGCCCGACCG<br>TGTTATATGTGAGCGGCGGTAACACGCAGGTGATTGCCTACAGCGAACATCGTTA<br>TCGTATCTTTGGTGAAACCATCGATATTGCCGTGGGTAATTGCCTGGATCGTTTT<br>GCGCGTGTGCTGAAAATTTCGAACGATCCGAGCCCGGGCTACAACATTGAACAGA<br>TGGCCAAACGCGGCAAAAAATTAGTTGAACTGCCGTACACCGTGAAAGGTATGGA<br>TGTGAGCTTTTCGGGCATCCTGAGCTTCATTGAAGATGTTGCCCATCGCATGCTG<br>GCCACGGGCGAGTGTACCCCGGAAGATCTGTGTTTCAGCCTGCAGGAAACCGTGT<br>TTGCCATGCTGGTGGAGATCACCGAACGCGCGATGGCACATTGTGGCTCCCAGGA<br>AGCCCTGATTGTGGGTGGCGTGGGTTGTAATGTGCGTCTGCAGGAAATGATGGCC<br>ACCATGTGCCAGGAACGTGGTGCCCGCCTGTTTGCGACCGATGAACGCTTCTGTA<br>TTGACAATGGTGCGATGATTGCCCAGGCCGGCTGGGAAATGTTTCGTGCCGGTCA<br>CCGTACCCCGTTAAGTGATTCTGGCGTTACCCAGCGTTATCGCACGGATGAAGTG<br>GAGGTGACCTGGCGCGATTAACTCTAGGATCCTCTAGAAATTTTGTTTAACTTTA<br>AGAAGGAGATATAACCATGGCGGCGGCCCGCGCCACCACGCCGGCCGATGGCGAA<br>GAGCCGGCCCCGGAAGCCGAGGCGCTGGCCGCAGCCCGTGAACGCAGCAGCCGCT<br>TCCTGAGCGGCCTGGAACTGGTGAAACAGGGTGCCGAGGCGCGCGTGTTCCGTGG<br>CCGCTTTCAGGGCCGCGCGGCGGTGATCAAACACCGCTTCCCGAAAGGCTACCGT<br>CACCCGGCGCTGGAAGCGCGCTTAGGCCGTCGCCGTACGGTGCAGGAAGCCCGCG<br>CGCTGTTACGCTGTCGCCGCGCGGGCATTAGCGCCCCGGTTGTGTTTTTTGTGGA<br>TTATGCCAGCAACTGCCTGTATATGGAAGAAATTGAAGGCTCGGTGACCGTTCGC<br>GATTATATTCAGAGCACGATGGAAACGGAAAAAACGCCGCAGGGTCTGTCCAACT<br>TAGCCAAAACCATTGGTCAGGTTTTAGCCCGCATGCACGATGAAGACCTGATTCA<br>TGGTGATCTGACCACCAGCAACATGCTGCTGAAACCGCCGCTGGAACAGCTGAAC<br>ATTGTGTTAATTGACTTTGGTCTGAGCTTCATTTCGGCCCTGCCGGAAGATAAAG<br>GCGTGGACTTATATGTTCTGGAAAAAGCCTTCCTGAGCACCCATCCGAACACCGA<br>AACGGTGTTTGAAGCCTTTCTGAAAAGCTACAGCACCAGCTCGAAAAAAGCCCGT<br>CCGGTGCTGAAAAAATTAGATGAAGTGCGCCTGCGCGGTCGTAAACGCAGCATGG<br>TTGGCTAACTAGAGAGCTCATTTTGTTTAACTTTAAGAAGGAGATATAAATATGC<br>AGCTGACCCATCAGCTGGACCTGTTTCCGGAATGCCGCGTGACCTTACTGTTATT<br>TAAAGATGTTAAAAATGCGGGCGATCTGCGTCGCAAAGCGATGGAAGGCACCATC<br>GATGGTAGCCTGATTAATCCGACCGTGATTGTTGATCCGTTTCAGATTCTGGTGG<br>CGGCCAACAAAGCGGTTCACTTATACAAACTGGGTAAAATGAAAACCCGCACGCT<br>GAGCACCGAAATTATTTTCAACTTAAGCCCGAATAACAATATTAGCGAAGCCCTG<br>AAAAAATTTGGTATCAGCGCGAATGACACCAGCATTCTGATTGTTTACATTGAAG<br>AGGGTGAAAAACAAATTAATCAGGAATACCTGATTAGCCAAGTGGAAGGTCATCA<br>GGTTTCTCTGAAAAATCTGCCGGAAATCATGAACATTACGGAAGTGAAAAAAATT<br>TATAAATTAAGCTCGCAGGAAGAAAGTATTGGCACCCTGTTAGATGCGATCATTT<br>GTCGCATGAGCACCAAAGATGTGCTGTAACTCTAAGCTTAATAATTTTGTTTAAC<br>TTTAAGAAGGAGATATACCATGCGTGATGCGGATGCGGATGCAGGCGGTGGCGCC<br>GATGGCGGTGACGGCCGCGGTGGCCACAGCTGCCGCGGCGGCGTGGATACCGCCG<br>CGGCCCCGGCCGGTGGCGCGCCGCCGGCGCACGCGCCGGGTCCGGGCCGTGATGC<br>CGCGAGCGCGGCCCGCGGCAGCCGTATGCGCCCGCACATCTTCACCCTGAGCGTG<br>CCGTTCCCGACCCCGCTGGAAGCGGAAATCGCCCACGGTTCGCTGGCGCCGGATG<br>CCGAACCGCACCAGCGTGTGGTTGGTAAAGATCTGACCGTGTCGGGCCGTATCCT<br>GGTTGTGCGCTGGAAAGCCGAAGATTGTCGCCTGTTACGTATTTCGGTCATCAAC<br>TTTCTGGACCAGTTAAGCCTGGTGGTGCGCACCATGCAGCGCTTTGGTCCGCCGG<br>TTAGCCGCTAATAACTGCGGCCGCCGCTAGCTTTGTTTAACTTTAAGAAGGAGAT<br>ATAGTTATGGAACTGCTGGGTGAATACGTTGGTCAGGAAGGCAAACCGCAGAAAC<br>TGCGTGTGAGCTGTGAAGCGCCGGGTGATGGCGACCCGTTCCAGGGCCTGCTGAG<br>CGGCGTGGCCCAGATGAAAGATATGGTGACGGAATTATTCGATCCGCTGGTGCAG<br>GGTGAAGTGCAGCACCGCGTGGCGGCGGCCCCGGACGAAGATCTGGATGGTGATG<br>ATGAAGATGATGCGGAAGATGAAAATAACATTGATAACCGTACCAACTTCGATGG<br>TCCGAGCGCCAAACGCCCGAAAACCCCGTCGCACCATCACCATCACCATTAACTC<br>GAG

Supplementary Table 8 | Sequences for expression of LAGE3-his, GON7-his and OSGEP/LAGE3/GON7-his for NMR, SAXS and/or crystallogenesis experiments.

Accession	Description	Coverage	# Unique Peptides	# PSMs
Q9BXV9	EKC/KEOPS complex subunit GON7 [GON7_HUMAN]	91,51	10	212
Q9NPF4	Probable tRNA N6-adenosine threonylcarbamoyltransferase OS=Homo sapiens [OSGEP_HUMAN]	66,37	24	131
Q14657	EKC/KEOPS complex subunit LAGE3 OS=Homo sapiens [LAGE3_HUMAN]	74,13	11	54
Q9Y3C4	EKC/KEOPS complex subunit TPRKB OS=Homo sapiens [TPRKB_HUMAN]	21,03	3	4
Q96S44	TP53-regulating kinase OS=Homo sapiens [PRPK_HUMAN]	17,36	1	2

Supplementary Table 9 | Identification of human KEOPS subunits by mass spectrometry in size-exclusion chromatography C3 fraction (from Supplementary Figure 13b).

Supplementary Methods

Additional information for cell lines. Lymphoblastoid cell lines (LCLs) were obtained by Epstein-Barr virus (EBV) transformation of B cells from peripheral blood (performed at the Necker Imagine DNA biobank, Paris, France) and were cultured in suspension at 37°C with 7% CO_2 in RPMI-1640 supplemented with 10% FBS, glutamine, ITS, and 50 IU/ml P/S (ThermoFisher Sientific).

Additional information for tRNA extraction from human primary fibroblasts. Briefly, small RNAs were extracted from 10.10^6 fibroblasts using Macherey Nagel's Isolation of small and large RNA kit, mixed with 2ml of R0 buffer and then loaded onto AXR-80 columns (Macherey Nagel's Genomic and total RNA purification kit). After binding to the column, tRNAs were selectively eluted using KCl gradient starting with 1ml of 450mM KCl followed by 2ml of 500mM KCl. The tRNA enriched fractions were pooled and precipitated with isopropanol. The pellet was washed two times with 70% Ethanol and resuspended in 50µl of water.

Additional information for Mass spectrometry analysis of t^6A.
tRNA hydrolysis. tRNA was unfolded at 95°C for 5 min and quickly incubated in a bath of water and ice for 5 min. Two units of nuclease P1 was mixed with the tRNA and incubated at 42°C for 2 hours. Then 0.01 unit of phosphodiesterase from snake venom was added and incubated for 1 hour at 37°C. Finally, the mixture was supplemented with 0.5 unit of alkaline phosphatase and incubated 1 hour at 37°C. To remove the enzymes, nucleosides were filtered onto 10kDa centrifugal units (Microcon) and filtrates were lyophilized and stored at -20°C.
LC-MS/MS analysis of RNA nucleosides. MS analysis was performed with a Hypersil Gold aQ column, 1.9 µm particle size, 2.1x100 mm (ThermoFisher Scientific) connected to an Acquity H-Class UPLC-XevoTQS instrument (Waters). The most intense MRM transitions used for integration and quantification were m/z 413>281 for t^6A at 3 min retention time, 267>136 for adenosine at 2 min, 284>152 for guanosine at 2.3 min, 243>110 for uridine at 1.3 min and 244>112 for cytidine at 1 min.

Additional information for Immunoprecipitation. Briefly, 48 hrs after transient transfection, HEK293T cells were lysed in buffer composed of 50mM Tris-HCl, pH7, 500mM NaCl, 1% Triton. One mg of protein was incubated either with anti-V5 antibody for 1 hr, followed by a 30-min incubation with magnetic beads-coupled to Protein A/G or directly with magnetic beads coupled to a HA antibody for 30 minutes (Miltenyi Biotec). Immunoprecipitated proteins were isolated using µMACS® Separation Columns in a magnetic µMACS separator and subsequently eluted using 1X Laemmli buffer. Lysates and immunoprecipitated samples were subjected to immunoblot.

Additional information for Cell-Proliferation Assay. Podocytes were seeded in triplicate 6 days after lentiviral transduction into 96-well plates at a density of 6 000 cells per well and allowed to attach for 24 hrs. Cell proliferation was assessed with the CellTiter 96 Aqueous Non-Radioactive Cell Proliferation Assay (MTT) (Promega) according to the manufacturer's protocol after 1, 2, 3, 4, and 7 days of culture.

Additional information for Apoptosis assay. Levels of apoptosis in KD podocytes were assessed using Caspase 3/7 Green detection Reagent (C10423, ThermoFisher Scientific) according to the manufacturer's instructions. Cells were seeded in triplicates at a density of $12x10^3$cells per well into 96-well plates (655-892, SensoPlate 96 wells glass bottom microplates, Greiner Bio-One). After 48 hrs, cells were incubated with 2µM of the reagent in 1x PBS, 3% FBS for between 1 and 6 hrs. The Caspase-3 or 7 activity in apoptotic cells was measured with a spectrophotometer (Berthold) at an absorbance at 502nm and normalized to nuclear DAPI fluorescence intensity.

Additional information for HPG Protein synthesis assay (l-homopropargylglycine based). Rates of protein synthesis were assessed in KD podocytes using the Click-iT HPG Alexa Fluor 488 Protein Synthesis Assays (C10428, Thermo Fisher) according to manufacturer's instructions. Cells were seeded at a density of 15×10^3 cells per well. After an incubation of 2 hrs with the reagent, intensity was measured with a microplate reader (LB 941 TriStar Berthold Technologies) and normalized to nuclear DAPI fluorescence intensity.

Additional information for production and purification of human KEOPS subunits.
For NMR experiments. Expression of his-tagged LAGE3 was achieved in *E. coli* BL21 gold strain grown in rich 2xYT medium. After reaching the logarithmic phase, 0.5 mM of IPTG was added for 3 hours at 37°C,

then cells were harvested by centrifugation, resuspended in 35 mL of lysis buffer A (Tris-HCl 20 mM pH 7.5 + 200 mM NaCl + 5 mM 2-mercaptoethanol) and stored at -20°C. Cells were lysed by sonication and centrifuged at 20 000g for 30 minutes. As LAGE3 is mainly insoluble, the pellet was recovered and washed with the same lysis buffer A supplemented with 0.5% Triton X100. After 30 minutes of centrifugation at 20 000 g, the pellet was resuspended in lysis buffer A + 8 M urea and centrifuged one more time at 20 000g. Supernatant containing solubilized, unfolded LAGE3 was then loaded onto NiNTA (Qiagen) affinity chromatography column and step-eluted in buffer A + 8 M urea containing increasing concentration of imidazole.
Expression of ^{15}N-GON7 was achieved in *E. coli* BL21 gold strain grown in M69 ^{15}N minimum medium and triggered by adding 0.5 mM of IPTG for 4 hours. Cells were collected by centrifugation and pellet was resuspended in 35 mL of lysis buffer A and stored at -20°C. Cells were lysed by sonication and centrifuged at 20 000 g for 30 minutes. The soluble supernatant containing ^{15}N-GON7 was purified by affinity chromatography on NiNTA. Purest fractions were pooled, concentrated and loaded on size exclusion chromatography column (Superdex 75 16/30) equilibrated in MES buffer 20 mM, pH 6.5 + 200 mM NaCl + 5 mM 2-mercaptoethanol. 200 µM of pure ^{15}N-his-GON7 was used for recording ^{15}N-HSQC spectrum at 20°C on a Bruker Avance III 800 MHz.
Subcomplex LAGE3/^{15}N-GON7 was prepared by refolding 387 µL of 600 µM of denatured his-LAGE3 with 200 µL 784 µM of ^{15}N-GON7 and loading the mixture on an analytical superdex S200 10/30 size exclusion chromatography column equilibrated in MES buffer 20 mM, pH 6.5 + 200 mM NaCl + 5 mM 2-mercaptoethanol. Fraction containing the binary complex were pooled and concentrated to 64 µM for recording the ^{15}N-HSQC spectrum at 20°C on a Bruker Avance III 800 MHz.
To probe the folding of YRDC protein and the effect of the p.Ala84Val and p.Leu265del mutations, we collected 1D ^{1}H spectra of 70uM (for wild-type and p.Ala84Val) and 40 uM (p.Leu265del) proteins dissolved in 25mM Tris, pH 7.5, 200mM NaCl, 5mM beta-mercapto, and 95% H_2O/ 5% D_2O. Water suppression was achieved by the excitation sculpting method and the 1D spectra were collected at 298K on a 950 MHz Bruker Avance III HD spectrometer equipped with a cryoprobe.

For SAXS or Crystallogenesis experiments. Unlabeled GON7 was prepared according to the above protocol except that E. coli BL21 GOLD cells were grown in rich 2xYT medium. The subcomplex LAGE3/GON7 was prepared by using two monocistronic vectors (pET21aLAGE3 and pET24d GON7. *E. coli* BL21 GOLD cells were cotransformed by adding 50 ng of each plasmid. The transformed cells were grown in rich 2YT medium during 3 hours at 37°C. Cells were then harvested by centrifugation, suspended in 40 mL of lysis buffer A (20 mM Tris buffer pH 7.5 + 200 mM NaCl + 5 mM 2-mercaptoethanol) and stored at -20°C or directly used for purification. Cells were lysed on ice by three successive sonication cycles of 30 seconds using a Branson Sonifier and centrifuged at 20000 g for 30 minutes. Supernatant (i.e. soluble cell extract) was loaded on NiIDA silica resin (Macherey Nagel, Duren, Germany) and 40 mL of buffer A was applied as a washing step. Bound proteins were eluted using 3 fractions of 2 mL of buffer A supplemented with 100, 200 and 400 mM imidazole, respectively, with samples from each purification step being analyzed by SDSPAGE. We loaded the pooled fractions onto an analytical S75 size exclusion chromatography column. LAGE3 and GON7 co-eluted as a single peak at 59 mL. Eluted fractions were analyzed by SDS PAGE (**Supplementary Fig.12**). The subcomplex OSGEP/LAGE3/GON7 was prepared by using a polycistronic vector (namely "pET24a-BC25_hKEOPS_opt") designed to express the complete (i.e. 5 subunits) human KEOPS complex according to a previously published strategy[4]. Briefly, human KEOPS genes were codon-optimized for bacterial expression using EuGene software[5], and subcloned into a pET24a vector between the NdeI and XhoI restriction sites and ordered from Genscript (Piscataway, USA). Sequence of the polycistronic gene is shown in **Supplementary Table 8**. The co-expression of the KEOPS subunits was achieved in the *E. coli* Rosetta pLysS strain at 37°C for 3 hrs. Cells were then harvested by centrifugation, suspended in 40 mL of lysis buffer A (20 mM Tris buffer pH 7.5 + 200 mM NaCl + 5 mM 2-mercaptoethanol) and stored at -20°C or directly used for purification. Cells were lysed on ice by three successive sonication cycles of 30 seconds using a Branson Sonifier and centrifuged at 20000 g for 30 minutes. Supernatant (i.e. soluble cell extract) was loaded on NiIDA silica resin (Macherey Nagel, Duren, Germany) and 40 mL of buffer A was applied as a washing step. Bound proteins were eluted using 3 fractions of 2 mL of buffer A supplemented with 100, 200 and 400 mM imidazole, respectively, with samples from each purification step being analyzed by SDS-PAGE. Interestingly, we could only co-express and co-purify OSGEP, LAGE3 and GON7 on the NiIDA column. Indeed, SDS-PAGE analysis of the eluted fractions (**Supplementary Fig.13a**) showed the presence of OSGEP, LAGE3 and GON7, with barely detectable amounts of TP53RK and TPRKB (which is probably due to their low soluble expression levels in our polycistronic expression vector system). We loaded the pooled fractions onto an analytical S200 size exclusion chromatography column. As shown in **Supplementary Fig.13b**, OSGEP, LAGE3 and GON7

co-eluted as a single peak at 14.5 mL suggesting they are forming a complex. The identity of the three proteins in the eluted peak was confirmed by mass spectrometry (**Supplementary Table 9**).

Additional information on sequence analysis

Multiple sequence alignments with human YRDC, *Sulfolobus tokadaii* Sua5 and *Pyrococcus abysii* Sua5, *Escherichia coli* YrdC and GON7 family were realised using blosum62 matrix[6]. The 3D model was generated using the protein fold recognition protocols of modeller software based on one – and three- dimensional sequence profiles[7]. Structure-based alignment of a subset was performed using the ESPRIPT platform[8]. All structural figures were generated with Pymol (The PyMOL Molecular Graphics System, Version 2.0 Schrödinger, LLC). The hydrophobic surface creation was performed by PyMOL command to color protein molecules according to the Eisenberg hydrophobicity scale[9]. Structural comparisons was performed with the PDBeFold web server[10].

Additional information for production and purification of human WT YRDC and mutants

DNA sequences of human YRDC WT, A84V and del265 have been purchased at Genscript (Piscataway NJ, USA) and subcloned in the pET21d vector between NcoI and BamHI, giving expression plasmids BC47_YRDC-WT, BC48_YRDC-A84V and BC49_YRDC-del165 respectively (see enclosed sequences in **Supplementary Table 3**). Bacteria BL21 Gold have been transformed with 50 ng of plasmid and grown at 37°C in 800 mL of 2xYT rich medium supplemented with ampicillin. When bacteria started to reach exponential phase growth (OD_{600nm} = 0.4), cultures have been shifted to 30°C for one hour and protein expression was triggered by adding 0.5 mM of IPTG. Cultures have been incubated for 3 more hours at 30°C and harvested by centrifugation at 6000 g for 20 minutes. Bacterial pellets were resuspended 35 mL of in Tris-HCl 25 mM, pH 7.5 + 200 mM NaCl + 5 mM 2-mercaptoethanol.
Cell pellets have been lysed on ice by sonication using a Branson Sonifier (3 cycles of 30 seconds) and centrifuged at 20000 g for 30 minutes. Supernatant has been loaded on NiNTA resin (Qiagen) and purified by adding successive fraction of Tris-HCl 25 mM, pH 7.5 + 200 mM NaCl + 5 mM 2-mercaptoethanol supplemented with 20 mM, 40 mM, 60 mM, 100 mM, 200 mM and 400 mM of imidazole. Protein fractions eluted at 200 and 400 mM of imidazole were pooled and further purified on S75 (16/60) superdex column (GE Healtcare) elquilibrated with Tris-HCl 25 mM, pH 7.5 + 200 mM NaCl + 5 mM 2-mercaptoethanol. Purity was checked by SDS-PAGE and the purest fraction were pooled and concentrated by ultrafiltration. Finally, YRDC-WT could be concentrated up to 5 mg/mL, YRDC-A84V up to 3 mg/mL and YRDC-del265 up to 1 mg/mL.

Additional information for the activity assay of YRDC WT and mutants *in vitro*.

Activity of YRDC WT and mutants has been assayed in multi-well plate using the published malachite green method[11]. Pyrophosphatase (#I1643 from Sigma) was used as the coupling enzyme. The following solutions were prepared:
-Staining solution: 5 mL of Ammonium molybdate 4.2% (w/v) + 15 mL of malachite green 0.045 % (w/v) were mixed at room temperature for 30 minutes and filtered through 0.22 µm.
-Stop solution: NaAcetate 0.25 M pH4
-Pre-mix solution (for 4 reactions): 60 µL of ATP at 1.5 mM + 120 µL of $MgCl_2$ at 1.5 mM + 30 µL of $NaHCO_3$, at 100 mM + 60 µL of L-threonine at 1.5 mM + 2 µL of 1U/µL Pyrophosphatase + 288 µL of Tris-HCl 50 mM pH7.5. For the pre-mix solution, all of the mentioned stock solutions of substrates and Pyrophosphatase have been prepared in Tris-HCl 50 mM pH7.5.
-50 µL of stop solution were dispensed in enough wells of a microtiter plate held on ice.
At t=0, 140 µL of pre-mix was incubated with 10 µL of 1.4 mg/mL of YRDC WT or mutants (or 10 µL of Tris-HCl 50 mM pH7.5 for the pre-mix solution) and 20 µL were immediately mixed in 50 µL of stop solution. The remaining solution was incubated at 30°C and aliquots of 20 µL were mixed with 50 µL of stop solution at 10’, 20’, 30’, 60’ and 90’.
-60 µL of staining solution were added in each wells of the microtiter plate which was incubated at room temperature. For standard curve determination, concentrations of inorganic phosphate (5 to 50 µM) were assayed in parallel in the same microtiter plate
Absorbance at 620 nm was read after 15- and 30-minutes using a Tecan Infinite 200 PRO plate reader. Absorbance values were plotted versus time and each kinetic was fitted by linear regression using Excel software (Microsoft). Initial velocity (mAU/min) was calculated as the slope of each line. Each kinetic was recorded in triplicate.

Additional references

1. Schanda, P. & Brutscher, B. Very fast two-dimensional NMR spectroscopy for real-time investigation of dynamic events in proteins on the time scale of seconds. J Am Chem Soc. 127, 8014-8015 (2005)
2. Hopkins, J.B., Gillilan, R.E. & Skou, S. BioXTAS RAW: improvements to a free open-source program for small-angle X-ray scattering data reduction and analysis. J Appl Crystallogr. 50, 1545-1553 (2017)]
3. Hajizadeh NR, Franke D, Jeffries CM, Svergun DI. *Consensus Bayesian assessment of protein molecular mass from solution X-ray scattering data. Sci Rep*. 2018 8(1):7204)
4. Collinet, B. et al. Strategies for the structural analysis of multi-protein complexes: lessons from the 3D-Repertoire project. *J Struct Biol.* **175**, 147-158 (2011).
5. Gaspar, P., Oliveira, J.L., Frommlet, J., Santos, M.A. & Moura, G. EuGene: maximizing synthetic gene design for heterologous expression. *Bioinformatics.* **28**, 2683-2684 (2012).
6. Corpet, F. Multiple sequence alignment with hierarchical clustering. *Nucleic Acids Res.* **16**, 10881-10890 (1988).
7. Sali, A., Potterton, L., Yuan, F., van Vlijmen, H. & Karplus, M. Evaluation of comparative protein modeling by MODELLER. *Proteins.* **23**, 318-326 (1995).
8. Robert, X. & Gouet, P. Deciphering key features in protein structures with the new ENDscript server. *Nucleic Acids Res.* **42**, W320-324 (2014).
9. Eisenberg, D., Schwarz, E., Komaromy, M. & Wall, R. Analysis of membrane and surface protein sequences with the hydrophobic moment plot. *J Mol Biol.* **179**, 125-142 (1984).
10. Krissinel, E. & Henrick, K. Secondary-structure matching (SSM), a new tool for fast protein structure alignment in three dimensions. *Acta Crystallogr D Biol Crystallogr.* **60**, 2256-2268 (2004).
11. Sousa, E.H., Garay, P.A., Tinianow, J.N. & Gerber, N.C. Development of a spectrophotometric assay for cyclase activity. *Anal Biochem.* **348**, 57-63 (2006).
