# Supplementary material for: Defects in t6A tRNA modification due to GON7 and YRDC mutations lead to Galloway-Mowat syndrome
Source: Nat Commun. 2019 Sep 3;10:3967. doi: 10.1038/s41467-019-11951-x (PMC6722078; doi:10.1038/s41467-019-11951-x)
Supplement: Supplementary file 3 — Reporting Summary [file 41467_2019_11951_MOESM3_ESM.pdf]

nature research

Corresponding author(s): Géraldine Mollet and Herman van Tilbeurgh

Last updated by author(s): Aug 5, 2019

Reporting Summary

Nature Research wishes to improve the reproducibility of the work that we publish. This form provides structure for consistency and transparency in reporting. For further information on Nature Research policies, see Authors & Referees and the Editorial Policy Checklist.

Statistics

For all statistical analyses, confirm that the following items are present in the figure legend, table legend, main text, or Methods section.

n/a	Confirmed	
☐	☒	The exact sample size (*n*) for each experimental group/condition, given as a discrete number and unit of measurement
☐	☒	A statement on whether measurements were taken from distinct samples or whether the same sample was measured repeatedly
☐	☒	The statistical test(s) used AND whether they are one- or two-sided<br>*Only common tests should be described solely by name; describe more complex techniques in the Methods section.*
☐	☐	A description of all covariates tested
☐	☒	A description of any assumptions or corrections, such as tests of normality and adjustment for multiple comparisons
☐	☒	A full description of the statistical parameters including central tendency (e.g. means) or other basic estimates (e.g. regression coefficient) AND variation (e.g. standard deviation) or associated estimates of uncertainty (e.g. confidence intervals)
☐	☒	For null hypothesis testing, the test statistic (e.g. *F*, *t*, *r*) with confidence intervals, effect sizes, degrees of freedom and *P* value noted<br>*Give P values as exact values whenever suitable.*
☐	☐	For Bayesian analysis, information on the choice of priors and Markov chain Monte Carlo settings
☐	☐	For hierarchical and complex designs, identification of the appropriate level for tests and full reporting of outcomes
☐	☐	Estimates of effect sizes (e.g. Cohen's *d*, Pearson's *r*), indicating how they were calculated

Our web collection on statistics for biologists contains articles on many of the points above.

Software and code

Policy information about availability of computer code

Data collection	*Provide a description of all commercial, open source and custom code used to collect the data in this study, specifying the version used OR state that no software was used.*
Data analysis	Softwares used to analyze the data are mentionned in the Materials and Methods and Supplementary Methods sections, when applicable.

For manuscripts utilizing custom algorithms or software that are central to the research but not yet described in published literature, software must be made available to editors/reviewers. We strongly encourage code deposition in a community repository (e.g. GitHub). See the Nature Research guidelines for submitting code & software for further information.

Data

Policy information about availability of data

All manuscripts must include a data availability statement. This statement should provide the following information, where applicable:

- Accession codes, unique identifiers, or web links for publicly available datasets
- A list of figures that have associated raw data
- A description of any restrictions on data availability

The data generated during the current study are available from the corresponding authors upon reasonable request.

Field-specific reporting

Please select the one below that is the best fit for your research. If you are not sure, read the appropriate sections before making your selection.

☒ Life sciences ☐ Behavioural & social sciences ☐ Ecological, evolutionary & environmental sciences

October 2018

For a reference copy of the document with all sections, see nature.com/documents/nr-reporting-summary-flat.pdf

Life sciences study design

All studies must disclose on these points even when the disclosure is negative.

Sample size	The study described here is on a rare genetic disease. As stated in the main text, 14 individuals with the relevant disease phenotype were examined. No statistical methods were used to determine sample size.
Data exclusions	No data was excluded from the analyses.
Replication	To verify the reproducibility of the experimental findings, each experiment was performed a least three times. This is stated in the legend of each figure and supplementary figures.
Randomization	Not applicable.
Blinding	Investigators were not blinded.

Reporting for specific materials, systems and methods

We require information from authors about some types of materials, experimental systems and methods used in many studies. Here, indicate whether each material, system or method listed is relevant to your study. If you are not sure if a list item applies to your research, read the appropriate section before selecting a response.

Materials & experimental systems

n/a	Involved in the study	
☐	☒	Antibodies
☐	☒	Eukaryotic cell lines
☒	☐	Palaeontology
☒	☐	Animals and other organisms
☐	☒	Human research participants
☒	☐	Clinical data

Methods

n/a	Involved in the study	
☒	☐	ChIP-seq
☒	☐	Flow cytometry
☒	☐	MRI-based neuroimaging

Antibodies

Antibodies used	All antibodies are listed in the section "Antibodies and chemical compounds" in Materials and Methods.
Validation	Antibodies against LAGE3, GON7, OSGEP, TP53RK, TPRKB and YRDC were validated by the suppliers and we confirmed them by western blot using cell lysates expressing the corresponding human cDNA (transiently and stably expressed in an immortalized podocyte cell line) . For GON7 antibody, we also validated them on patient cell lines (patients have a mutation in GON7 leading to a truncated protein of 7 residues).

Eukaryotic cell lines

Policy information about cell lines

Cell line source(s)	Cell lines are described in the Materials and Methods and Supplementary Methods sections.
Authentication	The human immortalized podocyte cell line (AB8/13) was previously described (Saleem et al, 2002). HEK293T cells were received directly from the supplier (ATCC). Human primary fibroblasts and HEK293T cells have been authenticated by light microscopy but not by additional methods. Lymphoblastoid cell lines obtention is described in Supplementary Methods.
Mycoplasma contamination	All cell lines used in the course of this study were tested negative for mycoplasma contamination (this is stated in Supplementary Methods).
Commonly misidentified lines (See ICLAC register)	No cell line listed by ICLAC was used

Human research participants

Policy information about studies involving human research participants

Population characteristics	All informations related to participants are included in the Supplementary Table 1.

Recruitment | *Describe how participants were recruited. Outline any potential self-selection bias or other biases that may be present and how these are likely to impact results.*

Ethics oversight | It is stated in the Material and Methods section

Note that full information on the approval of the study protocol must also be provided in the manuscript.
